# Pentagalloyl Glucose-Targeted Inhibition of P-Glycoprotein and Re-Sensitization of Multidrug-Resistant Leukemic Cells (K562/ADR) to Doxorubicin: In Silico and Functional Studies

**DOI:** 10.3390/ph16091192

**Published:** 2023-08-22

**Authors:** Nathupakorn Dechsupa, Nopawit Khamto, Pornthip Chawapun, Sadanon Siriphong, Phattarawadee Innuan, Authaphinya Suwan, Thitiworada Luangsuep, Nichakorn Photilimthana, Witchayaporn Maita, Rossarin Thanacharttanatchaya, Padchanee Sangthong, Puttinan Meepowpan, Chatchanok Udomtanakunchai, Jiraporn Kantapan

**Affiliations:** 1 Molecular Imaging and Therapy Research Unit, Faculty of Associated Medical Sciences, Department of Radiologic Technology, Chiang Mai University, Chiang Mai 50200, Thailand; nathupakorn.d@cmu.ac.th (N.D.); phattarawadee_in@cmu.ac.th (P.I.); authaphinya_s@cmu.ac.th (A.S.); 2 Faculty of Associated Medical Sciences, Department of Radiologic Technology, Chiang Mai University, Chiang Mai 50200, Thailand; thitiworada.laungsuep@gmail.com (T.L.); nuniranam.lu@gmail.com (N.P.); giftwitchaya@gmail.com (W.M.); browkwa@gmail.com (R.T.); chatchanok.u@cmu.ac.th (C.U.); 3 Faculty of Science, Department of Chemistry, Chiang Mai University, Chiang Mai 50200, Thailandpornthip_chawapun@cmu.ac.th (P.C.); sadanon_siriphong@cmu.ac.th (S.S.); padchanee.sangthong@cmu.ac.th (P.S.); puttinan.m@cmu.ac.th (P.M.); 4 Graduate School, Chiang Mai University, Chiang Mai 50200, Thailand; 5 Interdisciplinary Program in Biotechnology, Graduate School, Chiang Mai University, Chiang Mai 50200, Thailand; 6 Center of Excellence in Materials Science and Technology, Chiang Mai University, Chiang Mai 50200, Thailand; 7 Center of Excellence for Innovation in Chemistry (PERCH-CIC), Faculty of Science, Chiang Mai University, Chiang Mai 50200, Thailand

**Keywords:** pentagalloyl glucose, P-glycoprotein, drug resistance, PGG, sensitizer, doxorubicin, combination therapy

## Abstract

Combining phytochemicals with chemotherapeutic drugs has demonstrated the potential to surmount drug resistance. In this paper, we explore the efficacy of pentagalloyl glucose (PGG) in modulating P-gp and reversing multidrug resistance (MDR) in drug-resistant leukemic cells (K562/ADR). The cytotoxicity of PGG was evaluated using a CCK-8 assay, and cell apoptosis was assessed using flow cytometry. Western blotting was used to analyze protein expression levels. P-glycoprotein (P-gp) activity was evaluated by monitoring the kinetics of P-gp-mediated efflux of pirarubicin (THP). Finally, molecular docking, molecular dynamics simulation, and molecular mechanics with generalized Born and surface area solvation (MM-GBSA) calculation were conducted to investigate drug–protein interactions. We found that PGG selectively induced cytotoxicity in K562/ADR cells and enhanced sensitivity to doxorubicin (DOX), indicating its potential as a reversal agent. PGG reduced the expression of P-gp and its gene transcript levels. Additionally, PGG inhibited P-gp-mediated efflux and increased intracellular drug accumulation in drug-resistant cells. Molecular dynamics simulations and MM-GBSA calculation provided insights into the binding affinity of PGG to P-gp, suggesting that PGG binds tightly to both the substrate and the ATP binding sites of P-gp. These findings support the potential of PGG to target P-gp, reverse drug resistance, and enhance the efficacy of anticancer therapies.

## 1. Introduction

The development of acquired drug resistance during continuous chemotherapeutic treatment poses a significant challenge to cancer treatment. This resistance often reduces the effectiveness of anticancer drugs and worsens patient outcomes [1]. A growing body of research suggests the upregulation of P-glycoprotein (P-gp), alternatively referred to as multidrug resistance protein 1 (MDR1), plays a substantial role in the development of drug resistance [2,3]. P-gp is a membrane transporter protein encoded by the MDR1 gene. The P-glycoprotein (P-gp) comprises two hydrophobic transmembrane domains (TMDs), with each TMD spanning the cell membrane and consisting of six helices. Additionally, inside the cellular context, two hydrophilic regions, known as nucleotide-binding domains (NBDs), have a high affinity for ATP (Figure S1). P-gp plays a critical role by utilizing energy from ATP hydrolysis to efflux various compounds from cancer cells, reducing their intracellular concentrations and leading to treatment failure [1,4]. Hence, it is imperative to develop strategies to overcome drug resistance mediated by P-gp in order to enhance the effectiveness of anticancer therapies. Several means of addressing this challenge are currently being explored. One such process involves using P-gp inhibitors, which can counteract the efflux activity of P-gp and enhance the intracellular concentration of anticancer drugs [5,6]. Combination therapy with non-toxic sensitizers, reversal agents, and chemotherapeutic drugs is also being studied as a potential strategy.

**Figure 1:**
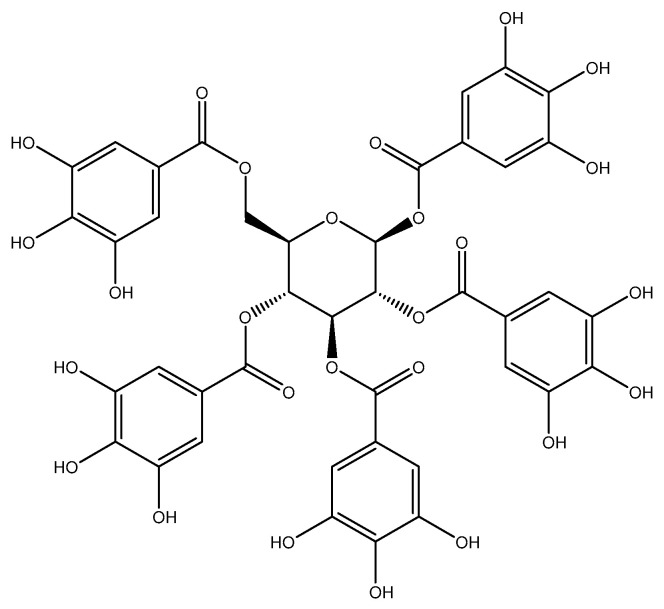
Chemical structure of pentagalloyl glucose (1,2,3,4,6-penta-*O*-galloyl-*β*-D-glucose, PGG).

The combination of treatment strategies incorporating several agents with distinct mechanisms of action has been demonstrated to be a more effective cancer therapy. This approach can reduce drug resistance and minimize the dosage of the chemotherapeutic drugs required in the treatment regimen, thereby circumventing significant adverse effects, all while preserving the therapeutic efficacy against cancer [7]. In cancer research, polyphenols have gained considerable attention due to their ability to influence various cellular processes that are involved in cancer development and progression [8,9]. Notably, polyphenols have shown promising results in overcoming drug-resistance mechanisms, including those mediated by P-gp [10,11]. Among polyphenols that exhibit MDR-reversing properties, curcumin has been reported to reverse MDR in P-gp overexpressing cancer cells in many cancers, including the human cervical carcinoma cell line (KB–V1) [12], the doxorubicin-resistant lung cancer cells (A549/DOX) [13], and the Adriamycin-resistant leukemic cell lines (K562/Adr) [14]. These studies showed that P-gp function and expression are both inhibited due to their inhibitory action. However, curcumin still has limitations in clinical investigation because of its poor water solubility, poor gastrointestinal absorption, restricted tissue distribution, quick metabolism, and subsequent fast elimination from the body [15]. This study focuses explicitly on pentagalloyl glucose (1,2,3,4,6-penta-*O*-galloyl-*β*-D-glucose, PGG), a moderately water-soluble polyphenolic compound derived from *Bouea macrophylla*. The chemical composition of PGG is shown in Figure 1, which is an esterification of glucose with five molecules of gallic acid, each with three hydroxyl groups on the benzene ring; the chemical structure of PGG appears to increase the inhibitory activity of the P-gp function [16,17].

PGG has demonstrated significant potential in combating cancer. Studies have revealed that PGG exerts its anti-cancer effect through its anti-proliferative, pro-apoptotic, and anti-metastatic effects against various cancer cells, such as breast, lung, colon, and prostate cancers [18,19,20,21]. Moreover, PGG has been found to sensitize cancer cells to different anti-cancer agents and radiotherapy, rendering them more susceptible to treatment [22,23,24]. Notably, one intriguing mechanism by which PGG demonstrates its anti-cancer properties is by downregulating the expression of MDR1. Studies have revealed that combining PGG with the chemotherapeutic drug fluorouracil (5-FU) results in a substantial reduction in the multidrug resistance protein 1 (MDR1) and the low-density lipoprotein receptor-related protein (LRP1) in aggressive HepG2 cells, indicating the potential to overcome resistance to 5-FU and enhance the efficacy of chemotherapy [25].

However, the precise role of PGG in multidrug resistance (MDR) remains unclear. Therefore, the present study aims to investigate the effects of PGG on MDR in the human leukemic K562 cell line, which is resistant to adriamycin (ADR) (K562/ADR). By utilizing the drug-resistant K562/ADR cells that overexpress P-gp, alongside the parental drug-sensitive K562 cells, this study aims to determine whether the combination of doxorubicin (DOX) and PGG, isolated from *Bouea macrophylla* seeds, possesses enhanced anti-tumor activities compared to the individual compounds. Additionally, we evaluate the molecular mechanisms by which PGG modulates P-gp expression and function. To produce greater detail and investigate the molecular interactions between PGG and P-gp, we employ in silico approaches, such as molecular docking, molecular dynamic simulation, and molecular mechanics with generalized Born and surface area solvation analyses (MM-GBSA). This investigation aims to shed light on the mechanisms underlying PGG’s action against MDR and to provide insights that are significant for the development of strategies for combatting drug resistance in leukemia. Ultimately, our findings may pave the way for developing novel therapeutic approaches that can enhance the efficacy of treatments in drug-resistant cancers and improve patient outcomes.

## 2. Results

### 2.1. PGG Enhances the Sensitivity of K562/ADR Cells to DOX

**Figure 2:**
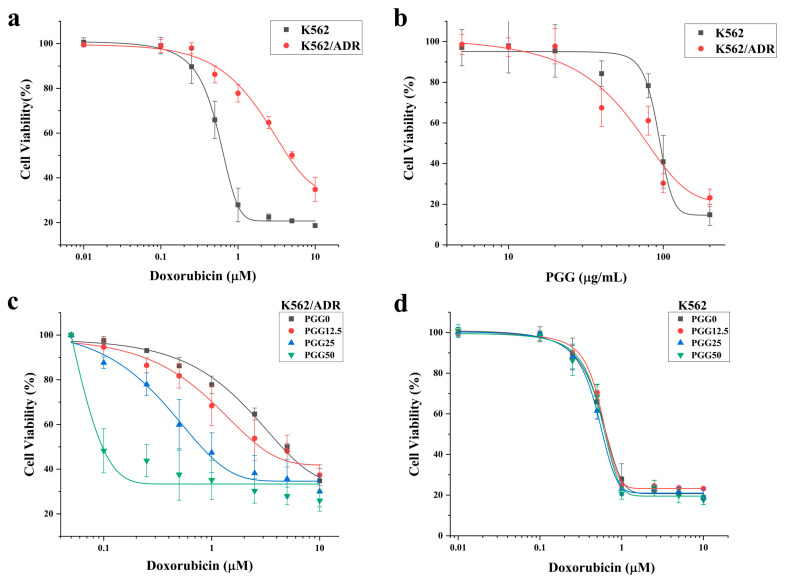
The cytotoxicity of Doxorubicin (DOX), PGG, and a combination of the two in DOX-resistant K562/ADR and DOX-sensitive K562 cells. (**a**) Cytotoxicity of DOX in K562 and K562/ADR cells. (**b**) Cytotoxicity of PGG in K562 and K562/ADR cells. (**c**) Effect of PGG on the cytotoxicity of DOX in K562/ADR cells. (**d**) Effect of PGG on the cytotoxicity of DOX in K562 cells.

First, we assessed the resistance index of drug-resistant K562/ADR cells compared to drug-sensitive K562 cells against PGG and doxorubicin (DOX) using the CCK-8 assay. The results shown in Figure 2a and Table 1 reveal that the K562/ADR cells exhibited significantly higher resistance to the cytotoxic effects of DOX compared to their parental K562 cell line, with a resistance index of 7.1. Conversely, PGG demonstrated a greater cytotoxicity towards K562/ADR cells compared to the K562 cells, with IC_50_ values of 77.94 ± 7.31 and 94.7 ± 3.84 µg/mL, respectively (Figure 2b). A combination treatment was used to evaluate the potential sensitizing effect of PGG on chemotherapeutic drugs in K562/ADR cells. Non-cytotoxic concentrations of PGG above the IC_50_ were employed to assess their impact on DOX’s half-maximal inhibitory concentration (IC_50_). The combination treatment displayed a dose-dependent inhibition of proliferation in drug-resistant K562/ADR cells. Notably, in the presence of 12.5, 25, and 50 µg/mL PGG, the inhibitory growth rates of DOX were dramatically increased. Consequently, PGG was observed to decrease the IC_50_ of DOX in K562/ADR cells and reached a reversal index of 23.7 at 50 µg/mL PGG (see Figure 2c and Table 1). PGG, however, did not affect the parental K562 cell line’s susceptibility to DOX (see Figure 2d). We draw our conclusion from these results that PGG can overcome the resistance of DOX in K562/ADR cells.

### 2.2. Effect of PGG on P-gp Protein Expression in K562/ADR Cells

**Figure 3:**
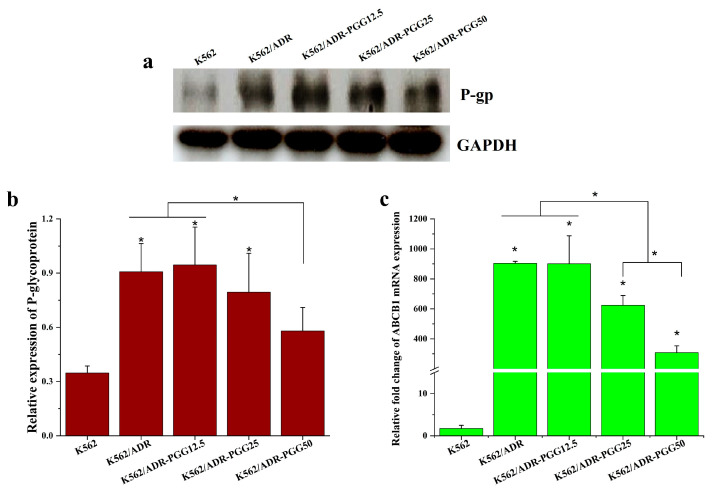
Evaluate the impact of PGG on P-gp expression in K562/ADR cells. K562/ADR cells were treated with PGG at 12.5, 25, and 50 µg/mL concentrations for 48 h. (**a**) P-gp levels were assessed using Western blotting with antibodies against the P-gp protein. (**b**) Protein band intensities were quantified using densitometry, and the protein expression level of P-gp was normalized with GAPDH, serving as an internal control. (**c**) MDR1 mRNA (*ABCB1*) expression was assessed using RT-qPCR. The results shown are the mean ± SD of three separate investigations. A one-way ANOVA and Tukey’s test were employed in the statistical analysis to ascertain the significance of mean differences. * *p* < 0.05 denotes a significant difference from the condition without PGG.

To investigate the mechanism underlying the reversal effect of PGG, we examined the impact of PGG on the expression of the MDR1 gene and P-gp protein in K562/ADR cells. After treating the cells with various concentrations of PGG (12.5, 25, and 50 µg/mL) for 48 h, we assessed the MDR1 gene and P-gp expression levels using RT-qPCR and Western blot assays. Our results show that the MDR1 gene and P-gp expression are significantly more prevalent in K562/ADR cells than in their parental K562 cells. We then analyzed how various PGG concentrations influenced the P-gp and MDR1 gene expression levels. The results demonstrate that the administration of PGG led to a significant reduction in the expression of the MDR1 gene and P-gp, particularly at the concentrations of 25 and 50 µg/mL, with the most pronounced effect observed at the highest concentration of 50 µg/mL (see Figure 3a–c). These results suggest that PGG exhibits a dose-dependent downregulation effect on P-gp expression, which may contribute to its ability to reverse drug resistance.

### 2.3. PGG Can Inhibit the Function of P-gp and Increases the Intracellular Accumulation of DOX

**Figure 4:**
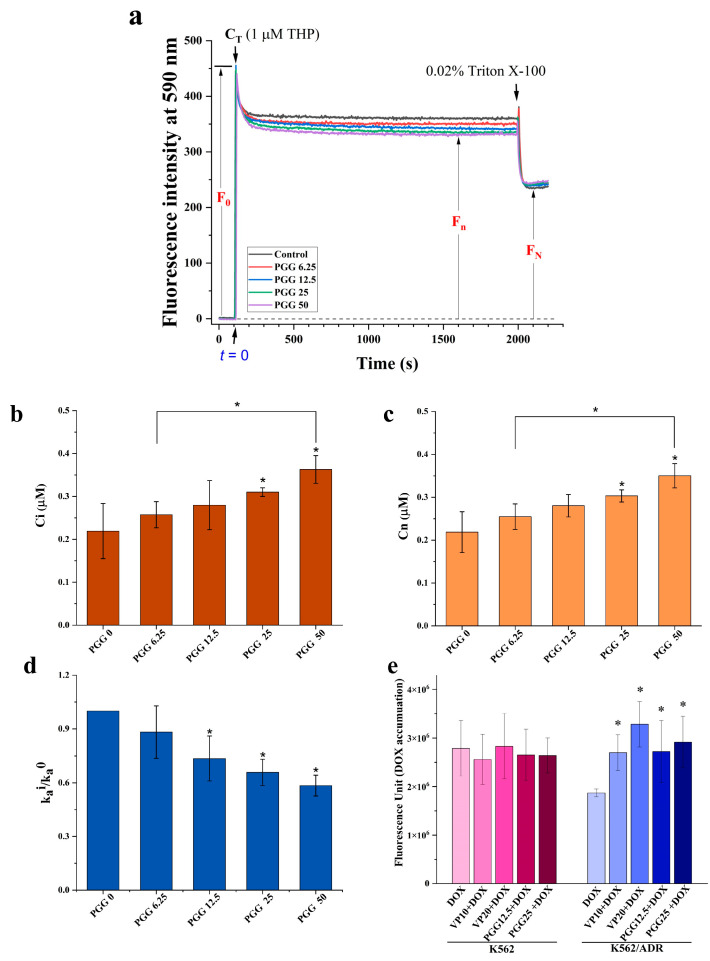
Effect of PGG on the accumulation of pirarubicin (THP), a P-gp substrate, in K562/ADR cells. (**a**) Representative kinetic profiles of THP influx and efflux in K562/ADR cells were recorded with and without PGG. The fluorescence intensity (F) at 590 nm (excitation at 480 nm) was continuously measured over time. Upon the addition of THP, the maximum intensity (F_max_) and the concentration of THP in the solution (C_T_) were determined. The F_0_ was calculated as (F_max_—background intensity). After adding 0.02% Triton X-100, a steady state was reached, leading to a subsequent decrease in the fluorescent intensity. (**b**) The intracellular concentration of THP (C_i_) was determined after incubating K562/ADR cells with different concentrations of PGG. (**c**) Overall nuclear concentration (C_n_) of THP at a steady state after the incubation of K562/ADR cells with various concentrations of PGG. (**d**) The ratio of k^i^_a_/k^0^_a_ represents the reduction in P-gp-mediated THP efflux. Three separate studies were performed in triplicate, and the results are provided as the mean ± SD. The statistical analysis was conducted with a one-way ANOVA and Tukey’s test. * *p* < 0.05 denotes a statistically significant difference compared to the condition without PGG. (**e**) The intracellular accumulation of DOX was assessed by measuring DOX fluorescence in both K562 and K562/ADR cells using a multimode microplate reader. Three separate studies were performed in triplicate, and the results are provided as the mean ± SD. The statistical analysis was conducted with a one-way ANOVA and Tukey’s test. * *p* < 0.05 denotes a statistically significant difference compared to the condition without PGG.

The findings depicted in Figure 3 demonstrate that treatment with PGG reduces the expression levels of P-gp. To further investigate this, we examined whether PGG inhibits the efflux function of P-gp. For this purpose, we utilized pirarubicin (THP), a doxorubicin derivative that exhibits minimal fluorescence interference and exhibits rapid intracellular accumulation compared to DOX. THP was employed as a substrate of P-gp to monitor cellular drug transport and the P-gp-protein-mediated drug efflux function in K562/ADR cells using spectrofluorometry. Drug-resistant K562/ADR cells incubated with varying doses of PGG show typical uptake kinetics of an anthracycline derivative, as shown in Figure 4a. The THP fluorescence at 590 nm in the cells was shown to diminish dose-dependently following the addition of PGG. This observation suggests that PGG has the potential to inhibit the P-gp-mediated efflux of THP in K562/ADR cells. We present the intracellular THP concentration (C_i_) and the overall concentrations of THP incorporated in the nucleus (C_n_) of K562/ADR cells at a steady state in various concentrations of PGG. The C_i_ and C_n_ values increased in K562/ADR cells when incubated with 6.25, 12.5, 25, and 50 µg/mL PGG compared to untreated cells (Figure 4b,c). Table 2 presents the kinetic parameters of THP transport in K562/ADR cells incubated with PGG and in untreated control cells. As expected, the kinetics of passive influx (V_+_) showed a significant increase in K562/ADR cells following incubation with 25 and 50 µg/mL PGG compared to the untreated control cells. In this study, we determined the P-gp-protein-mediated THP efflux rate (V_a_). The results reveal that the V_a_ and k_a_ values decreased in a dose-dependent manner in the presence of PGG. The ratio r = k^i^_a_ /k^0^_a_ was used to determine the degree to which PGG inhibited P-gp-mediated drug efflux; a value of 1 indicates that no inhibition of active efflux occurred, while 0 indicates that PGG completely prevented active efflux. Figure 4d illustrates the ratio values of k^i^_a_/k^0^_a_ in the K562/ADR cells incubated with PGG. The data reveal a notable decrease in the k^i^_a_/k^0^_a_ ratio values in PGG-treated cells at concentrations of 6.25, 12.5, 25, and 50 µg/mL compared to the untreated control cells. These findings strongly suggest the potential of PGG to inhibit the P-gp-mediated efflux of THP in K562/ADR cells. We then tested whether blocking P-gp’s efflux function with PGG may increase DOX accumulation intracellularly in K562/ADR cells. We performed an assay to determine the amount of DOX accumulated inside the cells to solve this problem. According to the data presented in Figure 4e, PGG increased the intracellular accumulation of DOX in a dose-dependent manner, with an effect similar to that of the particular P-gp inhibitor verapamil (VP).

### 2.4. PGG Augments DOX Treatment in Drug-Resistant K562/ADR Cells

**Figure 5:**
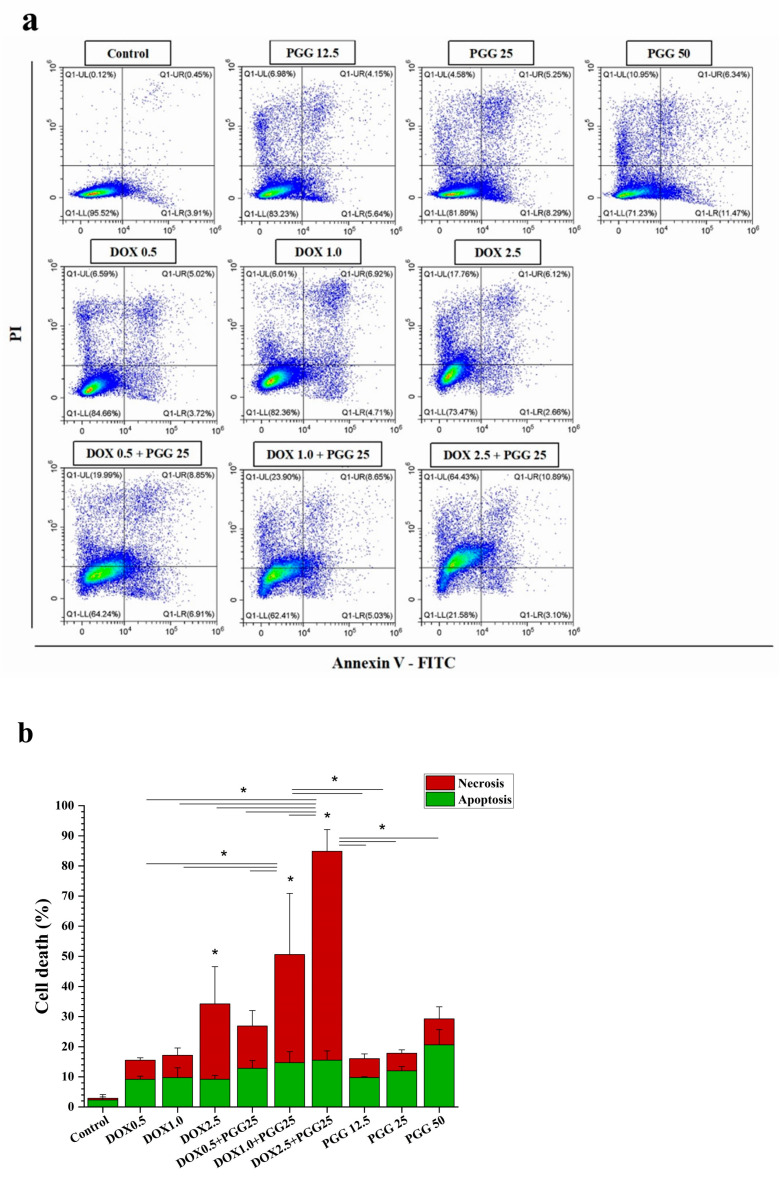
The induction of apoptosis in K562/ADR cells in response to DOX, PGG, or a combination of the two. K562/ADR cells were treated for 48 h with varying concentrations of DOX (0.5, 1.0, and 2.5 µM), PGG (12.5, 25, and 50 µg/mL), or a combination of 25 µg/mL PGG and varying concentrations of DOX. (**a**) Dot plot diagrams demonstrating the apoptosis responses of K562/ADR cells to the indicated compounds. (**b**) Flow cytometry quantification data show the proportion of total cell death in K562/ADR cells. Three separate experiments were conducted in triplicate, and the results are reported as the mean ± SD. One-way analysis of variance (ANOVA) and Tukey’s post hoc test were used for statistical analysis. * *p* < 0.05 indicates a significant difference compared to the control.

We assessed cell apoptosis to examine the underlying processes responsible for the antiproliferative effects of the combination of PGG and DOX. This assessment involved the utilization of double labeling with annexin V-FITC/PI, followed by subsequent analysis using flow cytometry. For the apoptosis induction experiments, we selected sub-lethal doses of DOX to inhibit cell growth by approximately 10% (IC_10_ = 0.5 µM), 20% (IC_20_ = 1.0 µM), and 30% (IC_30_ = 2.5 µM). After treating the cells for 48 h, we determined the percentages of apoptotic cells. The result show that, in the K562/ADR cells, both PGG and DOX induced cellular apoptosis in a dose-dependent manner as individual treatments. Interestingly, the treatment with a sub-lethal dose of DOX alone resulted in only a mild increase in the percentage of cell death. However, when combined with a non-toxic dosage of PGG, this sub-lethal dose of DOX significantly enhanced cell death compared to the single treatment (Figure 5a,b). The combined treatment resulted in an increase in cell death that was approximately two-to-three times higher than that produced by DOX alone. Furthermore, our results demonstrate that the PGG treatment groups exhibited a significant increase in cell apoptosis (Figure 5b). These findings suggest that the combination of PGG and DOX synergistically enhances the apoptotic response in K562/ADR cells, indicating a potential mechanism for the anti-proliferative effects observed with this combination treatment.

### 2.5. Molecular Docking

The binding poses of ligands in the P-gp binding sites were calculated using molecular docking. The protocol for molecular docking was validated by redocking a co-crystallized ligand into the binding site and then comparing the simulated configurations to the native crystallized structure. The results show similar binding poses for the docked and native ligands in both the substrate and ATP binding sites, as shown in the Supplementary Materials Figures S2 and S3. The RMSD value for tariquidar at the substrate binding site was found at 1.465 Å, and ATP at the ATP binding site was obtained at 0.766 Å. Additionally, the analysis of the protein–ligand interaction profiles of the docked and native ligands indicated a similar profile, suggesting a good docking protocol.

In this study, PGG, a candidate P-gp inhibitor, was docked into the substrate and ATP binding sites of P-gp. The binding ability of this compound was compared with that of standard inhibitors, verapamil and tariquidar. As a result, PGG was found to accommodate the substrate binding sites and ATP binding sites with binding energies of −9.3 and −9.5 kcal/mol, respectively. The binding energy from molecular docking suggested that PGG was tightly bound to both binding sites of P-gp. The binding poses were carefully selected and used as the initial structures for the molecular dynamics simulations.

### 2.6. Molecular Dynamics Simulation

**Figure 6:**
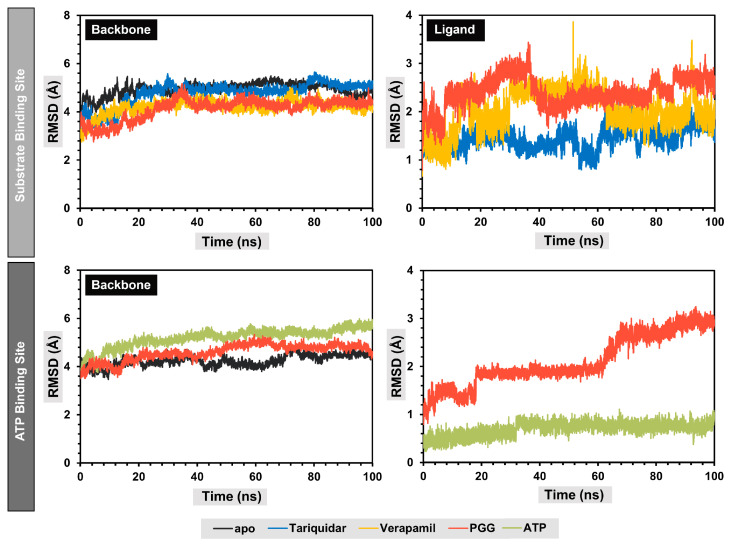
Root-mean-square deviation (RMSD) of protein backbones and ligands during molecular dynamics simulations.

A molecular dynamics simulation was used to assess the dynamics and stability of the protein–ligand complexes. To avoid the false positive control and increase reproducibility and reliability, according to the publication of Knapp, B. et al. [26], we performed six replica copies in each protein–ligand complex and three replicas were selected for visualization. For the discussion, only one replica was selected for analysis; for more details about the replicas, see Supplementary Figures S5–S9. The stability of the complexes was assessed by monitoring the root-mean-square deviation (RMSD). A fluctuation in the RMSD value of less than 2 Å indicated stable complexes. Consequently, the protein backbones of both the apo and holo forms of the complexes reached a stable state after 30 ns, with RMSD values at approximately 4 Å, as depicted in Figure 6.

Concerning the substrate binding site, it was observed that the protein backbones of the holo forms exhibited marginally reduced RMSD values compared to the apo forms, suggesting that the interaction between the ligand and the binding site of P-gp enhanced the structural stability of the protein folding. The secondary structure of the protein backbones was also similar to the apo form. The analysis of the RMSD values of the ligands indicated that the binding poses of the ligands in the substrate binding site changed during the production stages compared to the initial structures, with RMSD values around 1.7, 1.9, and 2.6 Å for native ligand tariquidar, verapamil, and PGG, respectively. At the ATP binding site, the protein backbone showed slightly higher RMSD values, indicating structural changes during the simulation. Additionally, PGG exhibited fluctuations during the simulation at approximately 20 and 60 ns, indicating conformational changes to more highly stable binding poses, which remained stable thereafter, with an RMSD value of approximately 3 Å. Meanwhile, the ATP remained stable throughout the simulation, with an RMSD of approximately 0.5 Å.

**Figure 7:**
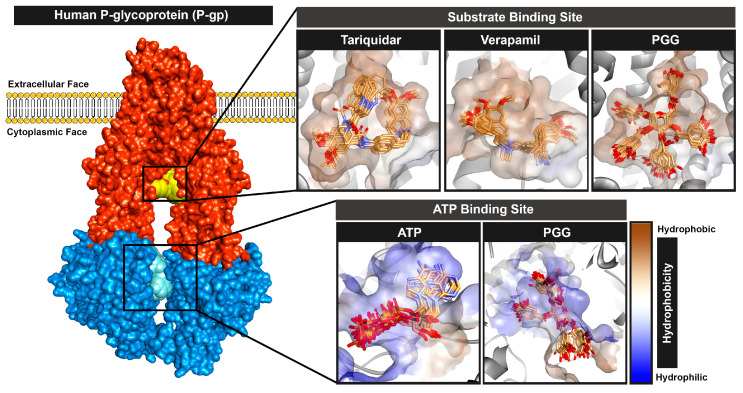
Binding poses of ligands of PGG and commercially available standards at the substrate and ATP binding sites during the molecular dynamics simulation.

Overall, all protein–ligand complexes reached a stable state during the 100 ns molecular dynamics simulations. The binding poses extracted from the lowest free energy according to free energy landscape (FEL) analysis with the range of 10 ns were stacked and are represented in Figure 7. The stack plots of the protein–ligand complexes showed slight conformational changes during the simulation, indicating that the complexes achieved equilibration. Furthermore, we confirmed the complex’s attainment of a stable state by conducting a cluster analysis, which demonstrated only one major cluster. In summary, PGG are bound stably to both binding sites of P-gp.

**Figure 8:**
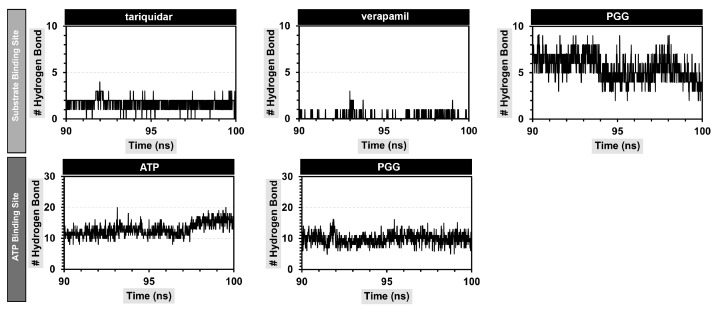
Number of hydrogen bonds between PGG and P-gp at the substrate and ATP binding sites.

The structure of PGG possessed a number of hydroxy groups, which can promote hydrogen bonding interactions with P-gp. A cut-off value of 3.5 Å was used to monitor the number of hydrogen bonds formed during the simulation. The results are shown in Figure 8. As a result, PGG formed stable hydrogen bonds with residues in a substrate-binding pocket, averaging 5.54 ± 1.37 bonds; this value was higher than those of the standard P-gp inhibitors tariquidar and verapamil, which average 1.70 ± 0.58 and 0.17 ± 0.40 bonds, respectively. When considering the ATP binding site, PGG formed a stable hydrogen bond of 8.59 ± 2.10 bonds, which was lower than ATP, with 12.93 ± 2.12 bonds. From this observation, it can be concluded that PGG primarily interacts with P-gp through hydrogen bonds.

The hydrogen bonds were further investigated by measuring the percentage of occupancy, as demonstrated in Table 3. At the substrate binding site, PGG primarily formed hydrogen bonds with the Y310, Q725, E875, and Q990 residues. The hydrogen bonding was found within a distance range of 2.032 to 2.698 Å. E875 exhibited the highest occupancy percentage at 109.09, representing the interaction of the hydroxyl group of PGG with the carboxylate group of P-gp. Regarding the ATP binding site, PGG formed hydrogen bonds with the Q441, Q438, E476, D555, D1171, and S1177 residues. Notably, the Q441, E476, E555, and D1171 residues demonstrated a high percentage of occupancy. Our observations suggest that the abundance of hydroxy and carbonyl ester groups in PGG promoted hydrogen bonding interactions between PGG and P-gp at both the substrate and ATP binding sites.

### 2.7. Calculating the Binding Free Energy

Molecular mechanics with the generalized Born and surface area (MM-GBSA) method was utilized to calculate the relative binding free energies. The binding energies calculated using this approach are generally more accurate than those obtained from the molecular docking technique. Although the substrate binding site showed a higher hydrophobic contribution than the ATP binding site, the internal dielectric constant of the substrate binding site was set to be lower than that of the ATP binding site. The binding free energy was calculated from the average of three replica copies of the complexes, as depicted in Table 4.

At the substrate binding site, our candidate P-gp inhibitor, PGG, demonstrated a binding free energy of −43.07 ± 4.53 kcal/mol, which was slightly higher than that of the commercially available inhibitors tariquidar. The binding free energy was primarily driven by van der Waals ($\Delta E_{\mathrm{vdw}}$) and electrostatic ($\Delta E_{\mathrm{elect}}$) interactions. The stronger van der Waals interaction was attributed to the higher molecular weight of PGG compared to verapamil and tariquidar. Additionally, the electrostatic energy was predominantly more negative than that of these commercial drugs, due to the higher number of hydrogen bonds. However, PGG exhibited a more positive polar solvation free energy ($\Delta G_{\mathrm{polar}}$). This was caused by the strong electrostatic interactions between PGG and solvent molecules, particularly through hydrogen bonding. Moreover, PGG demonstrated a higher conformational entropy (−TΔS) compared to the standard drugs verapamil and tariquidar, indicating a greater loss of the conformational degree of freedom. As a result, PGG exhibited higher binding free energy values than the standard drugs verapamil and tariquidar.

Regarding the binding of PGG at the ATP binding site, PGG showed a stronger negative binding energy than the native ligand ATP. The major binding ability was also supported by van der Waals and electrostatic interactions, as was the case for the substrate binding site, along with a high level of free energy of polar solvation. In contrast, ATP showed a smaller van der Waals interaction than PGG. The main interactions were generally driven by electrostatic interactions. This was due to the high polarity of ATP, which consisted of the nitrogenous base adenosine, ribose sugar, and triphosphate moieties. The electrostatic interaction of ATP was primarily encouraged by hydrogen bonds between the triphosphate group and protein, and it was also supported by the salt bridge and attractive charge. However, ATP had a high polar solvation energy, causing it to have a lower binding ability compared to PGG.

**Figure 9:**
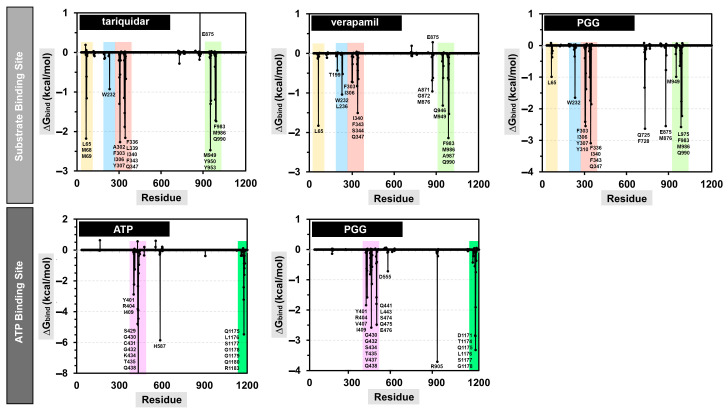
Energy decomposition per residue of the protein–ligand complexes at the substrate and ATP binding sites.

The examination of the energy distribution per residue provided insights into the specific amino acids that exerted a significant influence on the binding of the protein–ligand complexes. The decomposition results are shown in Figure 9, and only the residues that contributed to a binding energy lower than −0.5 kcal/mol are named.

**Figure 10:**
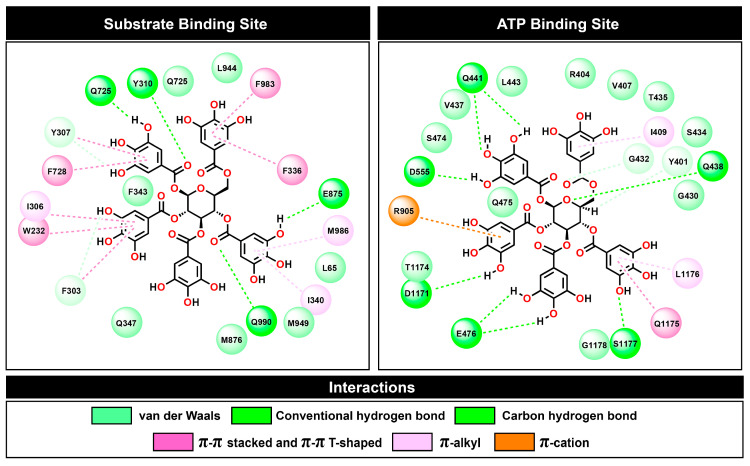
Two-dimensional plots of protein–ligand interactions between PGG and P-gp at the substrate and ATP binding sites.

At the substrate binding site, PGG showed a similar amino acid binding pattern to the commercially available drugs tariquidar and verapamil (in Figure 9, the same color indicates a similar pattern). This evidence indicates that PGG occupied the same site as those standard drugs. PGG interacted with the residues L65, W232, F303, I306, Y307, Y310, F336, I340, F343, Q347, Q725, F728, E875, M876, M949, L975, F983, M986, and Q990. The three phenylalanine residues F343, F728, and F952 mainly contributed to the binding ability with energies of −3.66, −3.49, and −3.38 kcal/mol, respectively, through the π–π stacked and π–π T-shaped interactions of aromatic rings on phenylalanine and PGG, as depicted in Figure 10. At this site, the hydrogen bonding slightly supported binding energy due to the high lipophilicity of the site. The decomposition suggested that the hydrogen-bond-contributing residues Y310, Q725, and Q990 showed decomposition energy ranges from −1.06 to −2.62 kcal/mol. Additionally, the binding ability was in part supported by the π–π interactions of aromatic amino acids, such as the W232, F303, Y307, F336, and F732 residues.

Regarding the ATP binding site, PGG interacted with residues similar to ATP. This suggests that PGG was stably bound to the ATP binding site. The main residues for PGG binding were Y401, R404, V407, I409, G430, G432, S434, T435, V437, Q438, Q441, L443, S474, Q475, E476, D555, R905, D1171, T1174, Q1175, L1176, S1177, and G1178. The residue R905 was the major contributor to the binding ability, with a free energy of −3.71 kcal/mol. The interaction occurred through π–cation interaction between the guanidine portion and the aromatic ring. In addition, the residues Q438, E476, T1174, Q1175, and L1176 showed strong binding free energy levels lower than −2 kcal/mol. The residues Q438 and E476 interacted through hydrogen bonds with energies of −2.58 and −2.48 kcal/mol, respectively. The residue T1174 interacted through a van der Waals interaction with an energy of −2.84 kcal/mol. Moreover, the residue Q1175 interacted through amide–π stacking between the carbonyl group and the aromatic ring of PGG, and L1176 interacted through a π–alkyl interaction. These residues contributed to binding free energy values of −2.86 and −3.32 kcal/mol, respectively.

According to the results from the in silico simulations, PGG was bound favorably to both the substrate and ATP binding sites. Our evidence suggests that PGG acts as a competitive inhibitor with the drug substrate, leading to a decrease in drug efflux from the cells, thereby improving the activity of drugs. In the case of the ATP binding site, PGG computationally demonstrated a greater binding free energy than ATP, which supported the observed decrease in the ATPase activity of P-gp [27]. Our in silico studies were in agreement with the in vitro assays.

## 3. Discussion

The emergence of drug resistance, particularly multidrug resistance (MDR), in cancer treatment poses a significant challenge that researchers and healthcare professionals are actively addressing. To improve the efficacy of anticancer therapies, it is crucial to develop treatment strategies to overcome drug resistance mediated by P-glycoprotein (P-gp). One approach that has attracted significant attention involves combining phytochemicals with low toxic profiles with conventional chemotherapeutic drugs. This combination therapy has shown the potential to induce synergistic effects, inhibit side effects, and overcome drug resistance [10,24,28]. In the present study, we demonstrated the ability of a phytochemical called PGG (previously isolated and purified from *Bouea macrophylla* seeds by our group) [23] to modulate the multidrug resistance protein P-gp. This study evaluated the MDR reversal activity of PGG in a drug-resistant leukemic cell line (K562/ADR) that overexpresses P-gp, compared to the drug-sensitive leukemic cell line K562. We conducted cytotoxicity tests to evaluate the effect of PGG on a drug-resistant leukemic cell line (K562/ADR) and a drug-sensitive leukemic cell line (K562). The results demonstrate that the IC_50_ value of PGG in the K562/ADR cells was 77.94 ± 7.31 µg/mL, which was lower than that in the K562 cells (IC_50_ = 94.7 ± 3.84 µg/mL). This finding indicates that PGG selectively induces cytotoxicity in the resistant cells, exhibiting a preference for targeting drug-resistant cells. Furthermore, we investigated the combination of PGG at non-toxic dosages (IC_10_, IC_20_, and IC_30_) with DOX and evaluated its effect on cell proliferation in K562/ADR cells. The combination treatment displayed a significantly stronger inhibitory effect on cell proliferation than the individual therapies. Notably, PGG alone did not exhibit substantial cytotoxicity at the selected concentrations. However, the reversal index reached up to 23.7 (Table 1), indicating that PGG effectively reversed drug resistance and enhanced the sensitivity of the resistant cells to DOX. These findings highlight the potential of PGG to be used as a reversal agent that can augment the toxicity of DOX in drug-resistant cells. Previous studies have also emphasized the augmented effects of PGG in conjunction with the conventional drug 5-FU in apoptosis induction. The combined treatment of PGG and 5-FU demonstrated to downregulate the expression of multidrug resistance protein 1 (MDR1) and low-density lipoprotein receptor-related protein 1 (LRP1), leading to the apoptotic induction of synergistic effects on the aggressive characteristics of HepG2 cells. Notably, this combination implies its potential to overcome 5-FU resistance [25].

P-glycoprotein (P-gp) is widely recognized as a critical player in developing drug resistance in various cell types. The overexpression of P-gp affects drug accumulation in cells by actively removing drugs in an ATP-dependent manner [29]. To improve treatment efficacy, suppressing P-gp expression and inhibiting its active function can enhance drug accumulation within cells. Thus, we hypothesized that PGG might exert a reversal effect by targeting P-gp expression and function. Our study investigated the association between PGG and the reversal effects on P-gp. The results reveal that PGG reduced the expression of P-gp and its gene transcript levels in a dose-dependent manner. We observed that K562/ADR cells overexpressed P-gp compared to K562 cells. Consequently, PGG did not significantly affect DOX sensitivity in the K562 model, likely due to the low expression of P-gp. To assess the impact of PGG on P-gp function, we examined the modulation of PGG on the kinetics of P-gp-mediated pumping and intracellular DOX accumulation.

To evaluate the kinetics of P-gp-mediated efflux in K562/ADR cells, we used the doxorubicin analog pirarubicin (THP) as a model anticancer drug. THP rapidly enters cells due to its relatively low pKa [30]. The kinetics of THP transport by drug-resistant K562/ADR cells at various PGG doses are depicted in Figure 4a. The accumulation of THP in K562/ADR cells was affected by the concentration of PGG, as evidenced by a decrease in fluorescence intensity at 590 nm. Additionally, we observed that the intracellular THP concentration (C_i_) and the overall concentrations of THP incorporated into the nucleus (C_n_) were significantly increased in PGG-treated K562/ADR cells compared to non-treated cells. These results suggest that PGG can enhance the intracellular THP concentration in K562/ADR cells. Moreover, the values of V_a_ (the apparent rate constant of THP efflux) and k_a_ (the rate constant for THP influx) decreased in a dose-dependent manner in PGG-incubated K562/ADR cells. Furthermore, the k^i^_a_/k^0^_a_ ratio values significantly decreased in cells incubated with various concentrations of PGG compared to non-incubated cells. These data indicate that PGG can inhibit P-gp-mediated efflux in K562/ADR cells [31]. Furthermore, we measured DOX accumulation within cells and found that PGG improved intracellular DOX concentration in a dose-dependent manner (Figure 4e). Together, these data raise the possibility that PGG might inhibit P-gp, resulting in more significant intracellular drug accumulation in K562/ADR cells. To further validate this finding, we conducted in silico molecular docking and molecular dynamics simulations, which provided valuable insights into the binding affinity of PGG towards P-gp at both the substrate and ATP binding sites. Our in silico study demonstrated that PGG exhibited tight and stable binding to P-gp, comparable to native ligands and standard inhibitors (verapamil and tariquidar). The interactions between PGG and the substrate binding site were predominantly driven by π interactions, with additional support from four hydrogen bonds. Furthermore, the binding of PGG to the ATP binding site was also favorable, facilitated by van der Waals forces, π–cation interactions, and hydrogen bonds. The robust binding ability of PGG can be attributed to its numerous phenolic portions or galloyl groups, which possess large hydroxy groups and aromatic rings that facilitate these interactions. Our in silico findings strongly suggest that PGG is a potent inhibitor of P-gp. PGG has a potent affinity for binding to the transmembrane (TMD) and nucleotide-binding domains (NBDs) of P-gp, sharing common binding sites with the anticancer drug and ATP in the corresponding functional regions. These shared binding sites may compromise P-gp’s efflux function by preventing it from binding to drugs or ATP. These molecular docking and molecular dynamics simulations provide essential insights into the binding mechanism of PGG with P-gp and support the suggestion that it is a promising candidate for further exploration in developing anticancer therapies.

Although the natural compound PGG used in this study is a potential compound for overcoming the multidrug resistance driven by the active efflux of antitumor drugs by the P-gp transporter, more research is needed to clarify the in vivo effects of PGG on multidrug resistance associated with P-gp in an animal model to establish with certainty the size of the dose necessary to achieve the same impact as the in vitro testing without causing significant adverse reactions. The researcher should thus consider their negative consequences at a high dosage. Recent research results showed that high doses of PGG (100 or 200 mg/kg/day) administered orally to mice did not have any adverse effects or cause weight loss in mice after seven days of therapy and that 200 mg/kg/day was the highest dose that could be tolerated; these results suggest that high doses of PGG are safe for mice [32]. PGG exhibits a propensity for lipids, polysaccharides, and proteins as evidenced by its slow clearance rate at 0.023 ± 0.012 h^−1^ and extended apparent elimination half-life (t_1/2_) around 38.66 ± 22.89 h after intraperitoneal administration to rats [33]. Using high-performance liquid chromatography coupled with quadrupole time-of-flight tandem mass spectrometry (HPLC-QTOF-MS), the in vivo metabolites of PGG in rat biofluids following a single intravenous administration of PGG (20 mg/kg) were identified. This study showed that PGG is initially converted to gallic acid in rats. The rat liver then converts gallic acid to its sulfated, glucuronidase, and methylated forms, eventually excreted from the body [34]. Furthermore, more information on the PGG-metabolized pharmacological activity and more detail of pharmacokinetics and their metabolism in the liver and gut in humans should be considered for further development of PGG as a potent P-gp inhibitor or anticancer drug.

## 4. Materials and Methods

### 4.1. Reagents

Abbkine Scientific (Biomedicine Park, Wuhan, China) was the vendor of the Cell Counting Kit-8 (CCK-8). Roswell Park Memorial Institute 1640 medium was acquired from the Caisson Lab (Smithfield, UT, USA). Gibco, Thermo Fisher Scientific (Waltham, MA, USA), provided cell culture supplies, including fetal bovine serum (FBS), penicillin, and streptomycin. Sigma-Aldrich (St. Louis, MO, USA) offered standard penta-O-galloyl-β-D-glucose hydrate (PGG). HiMedia Laboratories (Marg, Mumbai, India) supplied the PMSF and the protease inhibitor cocktail. Secondary antibodies labeled with horseradish peroxidase were purchased from Merck (Darmstadt, Germany).

### 4.2. PGG Isolation and Quantification

PGG was purified from the ethanolic extract of Bouea macrophylla seeds (MPSE) as previously described [23]. The lyophilized PGG pellets with a purity of 97% quantified using a High-Performance Liquid Chromatography (HPLC) method were collected for further experiments [35]. For the investigation, the stock solution of PGG (1 mg/mL) was prepared by dissolving PGG in 1 mL of high purity deionized water and sonicated for 30 min. The PGG solution was subsequently sterilized using a 0.22 µm membrane filter. Aliquots of the solution were prepared and stored at −20 °C until further use.

### 4.3. Cell Culture

This investigation employed human erythromyelogenous leukemia (K562) cells and their adriamycin-resistant counterparts (K562/ADR; cell lines that overexpress P-gp). The K562 and K562/ADR were made available by Laboratoire Chimie, Structures, Propriétés de Biomatériaux et d’Agents Thérapeutiques (CSPBAT), équipe NBD, Université Sorbonne Paris Nord, UMR-CNRS 7244, 1 rue de Chablis, 93000, Bobigny, France. The two cell lines were grown in RPMI 1640 medium mixed with 10% fetal bovine serum (FBS), 100 U/mL of penicillin, and streptomycin. The cells were cultivated in a 37 °C humidified, 5% CO_2_ environment. At a 5 × 10 ^5^ cells/mL density, the cells were dispersed and left for 24 h to divide and proliferate; this incubation period ensured that the cells had reached exponential growth before the start of the experiments.

### 4.4. Determining the Cytotoxicity and P-Glycoprotein Reversal Effect of PGG Using the CCK-8 Assay

To evaluate the inhibitory effect of PGG on cell proliferation, the Cell Counting Kit-8 (CCK-8) assay was applied. K562 and K562/ADR cells were used to evaluate the deleterious effects of PGG and DOX. The cells were inoculated at a density of 1 × 10^4^ cells per well in 96-well plates and treated with PGG (concentration range: 0–200 µg/mL) or DOX (concentration range: 0–10 µM). The plates were left to incubate for 48 h at 37 °C in a humidified atmosphere containing 5% CO_2_. Upon treatment, 10 µL of CCK-8 reagent was introduced to each well, and the cells were incubated for an additional 4 h. A multi-mode microplate reader (Molecular Devices SpectMax^®^ i3x, San Jose, CA, USA) was employed to measure the optical density at 450 nm. To determine cells’ relative viability, we normalized the optical density of the treated cells to that of the untreated cells. The average percentage of cell viability at each concentration was derived from experiments conducted in triplicate. These values were utilized to generate a dose–response curve using OriginPro software (Version 2023, Origin Lab, Northampton, MA, USA). The half-maximal inhibitory concentration (IC_50_) was determined from the dose–response curve.

To investigate the P-gp-reversing effect of PGG, we assessed the susceptibility to chemotherapy of P-gp-overexpressing resistant cancer cells to DOX (P-gp substrates). The effect of PGG on K562/ADR cells was evaluated by incubating the cells with fixed concentrations of PGG (12.5, 25, and 50 µg/mL) and varying concentrations of DOX (0–10 µM). Using the CCK-8 assay, cell growth inhibition was determined following a 48 h treatment. The reversal index was used to ascertain the efficacy of PGG in enhancing the cytotoxic effect of DOX on multidrug-resistant (MDR) cells.

### 4.5. P-Glycoprotein Expression by Western Blot Analysis

After treating K562/ADR cells with various concentrations of PGG for 48 h, the cells were harvested and lysed for a period of thirty minutes on ice using a CelLytic M tissue lysate solution (Sigma-Aldrich, St. Louis, MO, USA) containing 1% PMSF and a protease inhibitor cocktail. This lysis step facilitated protein extraction from the cells. The total protein concentrations in the lysates were quantified using the Bradford assay (Sigma-Aldrich, St. Louis, MO, USA). Twenty micrograms of protein samples were loaded and resolved on NUPAGE^TM^ 4–12% Bis-Tris Gels (Thermo Fisher Scientific, Waltham, MA, USA). The gel-separated proteins were deposited onto a PVDF membrane (Millipore, St. Louis, MI, USA). After 1 h of blocking with 5% nonfat dry milk in Tris-buffered saline with Tween, the membranes were incubated overnight at a dilution of 1:1000 with primary antibodies against rabbit polyclonal anti-P-gp IgG (Affinity Biosciences, Cincinnati, OH, USA) and mouse monoclonal anti-human GAPDH IgG (Affinity Biosciences, Cincinnati, OH, USA). Following the incubation of primary antibodies, the membranes underwent a washing procedure. They were subsequently exposed to secondary antibodies conjugated with the peroxidase enzyme horseradish at a dilution of 1:10,000 for 1 h at room temperature. The film pictures were acquired using ImageMaster 2D platinum software, version 5.0 (GE Healthcare Amersham Bioscience, Chicago, IL, USA), and the intensity of each band was quantified. To ensure accurate comparisons, the protein level was normalized to GAPDH, an internal control level.

### 4.6. Real-Time Quantitative PCR

K562 and K562/ADR cells were subjected to treatment with different doses of PGG (12.5, 25, and 50 µg/mL) for 48 h, and total RNA was extracted using the Nucleospin^®^ RNA Kit (Macherey-Nagel, Düren, Germany) per the instructions provided by the manufacturer. The extracted RNA was treated with rDNase (RNase-free) to remove genomic DNA contamination. The quantity of RNA was measured using a Nanodrop spectrophotometer (BioDrop™ μLITE, Cambridge, UK), and the A260/A280 ratio was determined to assess RNA purity. To generate complementary DNA (cDNA), RNA (0.5 µg) was reverse-transcribed using the ReverTra Ace™ qPCR RT Master Mix with a gDNA Remover (TOYOBO, Osaka, Japan) per the manufacturer’s instructions. A quantitative real-time polymerase chain reaction (qRT-PCR) was performed using THUNDERBIRD^TM^ Next SYBR^®^ qPCR Mix (TOYOBO, Osaka, Japan) and a CFX Connect™ real-time system (Bio-Rad, Hercules, CA, USA). The following are the primer sequences of the *ABCB1* (P-glycoprotein) primers:

Forward: 5′-GATAGGCTGGTTTGATGTGCACGATGTTGG-3′.

Reverse: 5′-CTGCCAAGACCTCTTCAGCTACTGCTCCAG-3′.

The following primer sequences were used for GAPDH (glyceraldehyde-3-phosphate dehydrogenase), which served as the internal control for normalizing the expression level of the target gene:

Forward: 5′-GTATCGTGGGAAGGACTCATGAC-3′.

Reverse: 5′-GAACATCATCCCTGCCTCTAC-3′.

The qRT-PCR conditions consisted of 40 cycles of initial denaturation at 95 °C for 60 s, denaturation at 95 °C for 10 s, annealing at 60.6 °C for 15 s, and extension at 72 °C for 30 s. Each reaction was conducted in triplicate. The cycle threshold (Ct) values of *ABCB1* were determined and normalized concerning the housekeeping gene GAPDH. The relative gene expression was calculated using the −2∆∆^Ct^ method, which represents the factor change in gene expression between the treatment and control groups [36].

### 4.7. Kinetic Study of P-gp-Mediated Efflux of THP in K562/ADR Cells Using the Spectrofluorometric Method

The P-glycoprotein (P-gp) function in living K562/ADR cells was assessed by investigating the kinetics of the active efflux of pirarubicin (THP), a P-gp substrate fluorescent agent. For this evaluation, a non-invasive functional spectrofluorometric method was used, as previously described [37,38,39]. This technique relies on measuring the quenching of THP fluorescence after its intercalation in DNA. In brief, K562/ADR cells (2.0 × 10^6^ cells) were incubated with different concentrations of PGG (6.25, 12.5, 25, and 50 µg/mL) in 2 mL of HEPES-Na^+^ buffer solution (containing 20 mM HEPES, 132 mM NaCl, 3.5 mM KCl, 1.0 mM CaCl_2_, and 0.5 mM MgCl_2_) supplemented with 5 mM glucose at a pH of 7.25. The incubation was conducted in 1 cm quartz cuvettes with vigorous stirring at 37 °C. Afterward, THP (1 µM) was introduced into the system; in the initial state (t = 0), the fluorescence intensity of free THP in the extracellular medium (C_T_) was equivalent to 1 µM THP, denoted as F_0_. As THP is taken up by the cells and intercalated into the DNA, its fluorescence is extinguished. A time-dependent decline in the THP fluorescence intensity was observed. When the steady state was reached (approximately 1600 s), the concentration of free THP in the extracellular medium and cytosol was equal, and the fluorescence intensity was denoted as F_n_. The concentration of THP incorporated in the nucleus (C_n_) was calculated as C_n_ = C_T_(F_0_ − F_n_)/F_0_. The steady-state fluorescence intensity in the presence of PGG, a P-gp inhibitor, was equal to F_n(i)_, and the concentration of THP intercalated between the base pairs of DNA in the nucleus was calculated as C_n(i)_ = C_T_(F_0_ − F_n(i)_)/F_0_. Subsequently, the cell membranes were permeabilized with 0.02% Triton X-100 to attain a state of equilibrium, resulting in the fluorescence intensity denoted as F_N_. The aggregate concentration of THP intercalated in the DNA (C_N_) was determined using the formula C_N_ = C_T_(F_0_ − F_N_)/F_0_. The ability of PGG to inhibit P-gp function was determined by calculating the ratio k^i^_a_/k^0^_a_, where k^i^_a_ is the P-gp-mediated active efflux of THP in the presence of PGG and k^0^_a_ is the P-gp-mediated active efflux of THP in the absence of PGG. When the P-gp function is entirely inhibited, the ratio k^i^_a_/k^0^_a_ equals 0. In addition, several parameters were determined, such as the rate of THP passive uptake (V_+_), the passive influx coefficient (k_+_), the rate of the P-gp-mediated active efflux of THP (V_a_), and the active P-gp-mediated drug efflux coefficient (k_a_).

### 4.8. Assay for the Intracellular Accumulation of Doxorubicin

By quantifying the fluorescence intensity of doxorubicin (DOX), the intracellular accumulation of doxorubicin (DOX) was determined. At a density of 5 × 10^5^ cells per well, K562 or K562/ADR cells were seeded in 24-well plates. The cells were then pre-treated with either 12.5 or 25 µg/mL of PGG or 10 or 20 µM of verapamil (VP) and cultured at 37 °C with 5% CO_2_ for a period of 24 h. Subsequently, 10 µM of DOX was added to each well, and the cells were allowed to incubate for an additional 1 h at 37 °C. Following the incubation with DOX, the cells were harvested, washed twice with ice-cold PBS, and lysed with a lysis buffer (CelLytic™ M Cell Lysis, Sigma-Aldrich, St. Louis, MO, USA). The intracellular fluorescence intensity associated with DOX was finally measured using a multi-mode microplate reader (Molecular Devices SpectraMax^®^ i3x, San Jose, CA, USA). The fluorescence intensity was measured at excitation and emission wavelengths of 480 and 590 nm, respectively.

### 4.9. Apoptosis Assay with Annexin V-FITC/PI Double Staining

K562/ADR cells (2 × 10^5^ cells/mL) were exposed to DOX at IC_10_ (0.5 µM), IC_20_ (1.0 µM), and IC_30_ (2.5 µM) concentrations, or co-treated with DOX at the respective concentrations along with PGG at 25 µg/mL for 48 h. Following the 48 h treatment, the cells were collected and rinsed with cold PBS (pH 7.4). The harvested cells were subjected to co-staining with Annexin V-FITC and PI for a duration of 20 min at room temperature under light-free conditions, following the provided instructions from the manufacturer. Flow cytometry immediately examined the labeled cells (CytoFLEX, Beckman Coulter, Brea, CA, USA). The data were then processed and analyzed with FlowJo^TM^ software version 10 (Becton, Dickinson & Company, Buena, NJ, USA) to ascertain the extent of the death of cells.

### 4.10. Molecular Docking

Molecular docking simulations were conducted using Autodock Vina [40]. The protein structures of human P-glycoprotein (P-gp) in complex with the inhibitor tariquidar (PDB ID: 7A6E) [41] and human P-glycoprotein in complex with ATP (PDB ID: 6C0V) [42] were retrieved from the Protein Data Bank (RCSB). Protein structures were prepared using the BIOVIA Discovery Studio visualizer 2021 [43] by removing co-crystalized ligands, ions, and water molecules. The missing residues and loop segments were modeled using the online tool SWISSMODEL [44]. The standard protonation states of the amino acids were calculated at a pH of 7.4 using the ProPka Server [45]. The 3D structures of the ligands PGG and verapamil were established using Chem3D software and minimized in terms of energy using the molecular mechanics force field (MM2). For tariquidar and ATP, the structures obtained from the co-crystallization were used. For the 3D structures of the ligands, see Supplementary Figure S4. The structures then underwent geometrical optimization using density functional theory (DFT) calculations with B3LYP/6-311++G (d,p) as the basis set via the Gaussian16 package [46]. Protein and ligand structures were converted to the pdbqt format using the AutoDock Tools 1.5.6 package [47]. The docking protocol was validated by redocking a co-crystalized ligand tariquidar to the substrate-binding pocket at the following coordinates: X = 161.296, Y = 159.843, and Z = 157.564, with a size of 40 × 40 × 40 for the X, Y, and Z coordinates. The ligand ID: R1H1301 was used as reference to compare RMSD value. Similarly, ATP was redocked to the ATP binding site at the following coordinates: X = 172.921, Y = 190.300, and Z = 132.053, with a size of 25 × 25 × 25 for the X, Y, and Z coordinates. The grid spacing was set as 0.375 Å for both proteins. The binding poses of the ligands were carefully selected for further investigation through molecular dynamics simulations based on protein–ligand interactions and binding energy profiles.

### 4.11. Molecular Dynamics Simulation

All molecular dynamics simulations were conducted on HPE Cray EX235n supercomputer with AMD EPYC 7713 CPUs. The simulations were accelerated by NVIDIA A100 SXM4 40GB GPUs operating under the HPE Cray operating system. Protein and ligand topologies were prepared using the AmberTools22 package [48]. The protein structure was parameterized using an AMBERff19SB force field [49]. The ligand charges were calculated at the theoretical level of Austin Model 1-bond charge corrections (AM1-BCC) via the antechamber, as implemented in AmberTools22. The general AMBER force field 2 (GAFF2) was used to provide atom types and atom parameters. The complexes were prepared using the tleap function by placing them in the octahedral box with a spacing distance of 10 Å from the protein surface. The complexes were further solvated by water molecules using the TIP3P model. Sodium (Na^+^) and chloride (Clˉ) ions were added to the system for neutralization with a concentration of 0.15 M. The AMBER topologies were converted to GROMACS topologies using the Python script ParmEd. All molecular dynamics simulations were performed using GROMACS (2022 version) on a GPU [50]. The energy of protein–ligand complexes were minimized with the steepest descent algorithm and a ten kJ/mol/nm tolerance. The complexes were additionally equilibrated by restricting the number of particles, volume, and temperature (NVT), followed by a 1000 ps ensemble with a constant number of particles, pressure, and temperature (NPT). To monitor the system’s equilibration, thermodynamic parameters, which include temperature, pressure, potential energy, and density, were observed to ensure adequate equilibration. The particle mesh Ewald (PME) method with a cut-off value of 12 Å was used to calculate long-range electrostatic interactions. The modified Berendsen thermostat [51] and Parrinello–Rahman barostat [52] were utilized for the temperature and pressure couplings, respectively. Last but not least, molecular dynamics simulations were run for 100 ns at 310 K and 1 bar of atmospheric pressure. All protein–ligand complexes were performed for six replica copies with identical initial structure. The initial velocity of atoms in each replica was randomized at 310 K for Maxwell distribution with the random seed. The trajectory was captured every 10 ps. The dynamics of the systems were monitored using the root-mean-square deviation (RMSD) calculated with the gmx_rms function. Hydrogen bonding formations were monitored using the gmx_hbond command implemented in the GROMACS packages and analyzed using the hydrogen bond analysis function in the VMD software [53].

The simulated trajectory was calculated and diagonalized for their covariance matrix using gmx_covar command followed by analyzing the eigenvectors using gmx_anaeig module. Free energy landscape (FEL) was calculated using gmx_sham command. The obtained matrix file was converted to eps file using gmx xpm2ps command and then converted to dat format using python script xpm2txt.py. The plot of FEL was achieved on Origin 2022 software.

### 4.12. Calculation of Binding Free Energy

Binding free energies were calculated using the gmx_MMPBSA packages [54], employing the molecular mechanics with generalized Born and surface area solvation (MM-GBSA) approach. The modified GB model 2 (GB-OBC2, igb = 5), developed by A. Onufriev, D. et al., was used as a solvation model [55]. In total, 1000 snapshots extracted from the lowest free energy region based on FEL analysis were used for the calculations. The dielectric constants parameters of the solute (intdiel) were defined as 1 and 2 for the substrate and ATP binding sites, respectively, along with a solvent (extdiel) value of 78.5 for both sites. The salt concentration was set at 0.15 M, and the solvent probe radius was defined as 1.4 Å. The binding free energy was calculated using the following equation:

(1) $$\Delta G_{\mathrm{bind}}=\Delta H-T\Delta S$$

(2) $$\Delta H=\Delta E_{\mathrm{MM}}+\Delta G_{\mathrm{sol}}$$

(3) $$\Delta E_{\mathrm{MM}}=\Delta E_{\mathrm{vdw}}+\Delta E_{\mathrm{elect}}$$

(4) $$\Delta G_{\mathrm{sol}}=\Delta G_{\mathrm{polar}}+\Delta G_{\mathrm{nonpolar}}$$

The total binding free energy ($\Delta G_{\mathrm{bind}}$) was calculated as the sum of the enthalpy of binding (∆H) and the conformational entropy after ligand binding (−TΔS). ∆H can be calculated from the changes in the molecular mechanical energy in the gas phase ($\Delta E_{\mathrm{MM}}$) and the free energy of solvation $(\Delta G_{\mathrm{sol}}).$ The potential energy was calculated based on the molecular mechanics force field to provide van der Waals ($\Delta E_{\mathrm{vdw}}$) and electrostatic ($\Delta E_{\mathrm{elect}}$) energies. The Gibbs free energy of solvation ($\Delta G_{\mathrm{sol}}$) was computed from the contribution of polar ($\Delta G_{\mathrm{polar}}$) and non-polar ($\Delta G_{\mathrm{nonpolar}}$) terms, which were calculated based on the generalized Born (GB) and the solvent accessibility surface area (SASA), respectively. The entropic component (−TΔS) was determined using the interaction entropy (IE) approach. All results for binding free energy were analyzed using the gmx_MMPBSA_ana program as embedded in the gmx_MMPBSA package. 

### 4.13. Statistical Analysis

The results of at least three separate investigations are averaged and then shown as mean ± standard deviations (SDs). A one-way analysis of variance (ANOVA) and post hoc analysis using the 2023 version of the OriginPro software (Origin Lab, Northampton, MA, USA) were employed to determine if there were significant differences in the means. Statistical significance was considered to occur when the *p*-value was less than 0.05.

## 5. Conclusions

In summary, this study highlighted the potential of PGG in targeting P-gp, reversing drug resistance, and enhancing the efficacy of anticancer therapies. PGG can inhibit P-gp-mediated efflux and increased intracellular drug accumulation in drug-resistant cells. Molecular docking and molecular dynamics simulations provided insights into the binding affinity of PGG to P-gp, suggesting that PGG binds tightly to both the substrate and ATP binding sites of P-gp. These findings support the potential of PGG to be used as a potent P-gp inhibitor in the development of anticancer drugs. However, further experimental studies and clinical trials are necessary to validate the efficacy and safety of PGG as a P-gp inhibitor and to explore its potential applications in cancer treatment.

## Figures and Tables

**Table 1:** Half-maximal inhibitory concentration (IC_50_) values and reversal effect of PGG in K562 and K562/ADR cells.

Drug	IC_50_ Mean ± SD ^a^		Resistance Index ^b^	Reversal Index ^c^
	K562	K562/ADR		
PGG	94.7 ± 3.84 µg/mL	77.94 ± 7.31 µg/mL		
DOX	0.64 ± 0.32 µM	4.54 ± 0.77 µM	7.1	1
DOX+PGG12.5	0.65 ± 0.16 µM	3.30 ± 1.99 µM	5.2	1.4
DOX+PGG25	0.60 ± 0.23 µM	0.95 ± 0.53 µM	1.5	4.7
DOX+PGG50	0.63 ± 0.13 µM	0.22 ± 0.03 µM	0.3	23.7

^a^ The IC_50_ values shown are the mean ± SD of three separate experiments performed in triplicate. ^b^ The index of resistance was calculated by dividing the IC_50_ values of K562/ADR cells in the presence or absence of PGG by the IC_50_ values of K562 cells without PGG. ^c^ The reversal index was calculated by dividing the resistance index values without PGG by the resistance index with or without PGG.

**Table 2:** Kinetic parameters of pirarubicin transport across K562/ADR cells following treatment with different concentrations of PGG.

PGG (μg/mL)	V_+_ (nM/s)	K_+_ × 10^−9^ L/Cell·s	V_a_ (nM/s)	K_a_ × 10^−9^ L/Cell·s
0	1.60 ± 0.2	0.98 ± 0.1	0.55 ± 0.03	2.9 ± 0.85
6.25	2.10 ± 0.3	1.13 ± 0.30	0.52 ± 0.02	2.3 ± 0.40
12.5	2.38 ± 0.3 ^a^	1.17 ± 0.05	0.49 ± 0.10	2.2 ± 0.56
25	2.52 ± 0.2 ^a^	1.28 ± 0.07 ^a^	0.42 ± 0.06 ^a^	2.04 ± 0.8 ^a^
50	2.70 ± 0.4 ^a^	1.36 ± 0.2 ^a^	0.35 ± 0.11 ^a^	1.06 ± 0.4 ^a^

Results are presented as the mean ± SD of triplicate samples. V_+_ represents the rate of pirarubicin uptake, while K_+_ represents the passive influx coefficient. V_a_ indicates the rate of P-gp-mediated active efflux of pirarubicin, and K_a_ represents the P-gp-mediated active efflux coefficient. (^a^ indicates statistical significance with *p* < 0.05 compared to the control).

**Table 3:** Percentage of occupancy of hydrogen bonds between PGG and P-gp.

No.	Hydrogen Bonding ^a^	Distance (Å) ^b^	Occupancy (%)
Substrate binding site			
1	E875-(C=O)-O ···· HO-PGG	2.432 ± 0.789	109.09
2	Y310-OH ···· O=C-PGG	2.032 ± 0.284	47.65
3	Q990-NH_2_ ···· O=C-PGG	2.159 ± 0.270	34.27
4	Q725-C=O ···· HO-PGG	2.698 ± 1.064	33.07
ATP binding site			
1	E476-(C=O)-O ···· HO-PGG	2.230 ± 0.845	216.88
2	D1171-(C=O)-O ···· HO-PGG	1.725 ± 0.152	111.59
3	D555-(C=O)-O ···· HO-PGG	1.703 ± 0.104	105.39
4	Q441-NH2 ···· O-PGG	2.131 ± 0.210	96.40
5	Q441-C=O ···· HO-PGG	1.935 ± 0.326	65.73
6	Q438-NH2 ···· O-PGG	2.158 ± 0.242	51.05
7	S1177-NH ···· O-PGG	2.892 ± 0.434	31.87

^a^ Only the hydrogen bonds with a criterion of the donor–acceptor angle > 120° and distance < 3.5 Å were observed. ^b^ Occupancy higher than 30% was demonstrated.

**Table 4:** Free energy terms of protein–ligand complexes calculated from MM-GBSA.

Compound	Free Energy (kcal/mol) ^a^					
	$\left(\Delta E_{\mathrm{vdw}}\right)$	$\left(\Delta E_{\mathrm{elect}}\right)$	$\left(\Delta G_{\mathrm{polar}}\right)$	$\left(\Delta G_{\mathrm{nonpolar}}\right)$	(−TΔS)	$\left(\Delta G_{\mathrm{bind}}\right)$
Substrate binding site						
Verapamil	−57.36 ± 1.51	−9.45 ± 0.86	31.87 ± 0.93	−8.21 ± 0.18	9.28 ± 0.62	−33.87 ± 1.77
Tariquidar	−76.09 ± 3.37	−25.31 ± 1.51	56.04 ± 1.10	−10.30 ± 0.48	10.02 ± 0.67	−45.64 ± 4.67
PGG	−85.52 ± 1.67	−87.62 ± 4.01	115.17 ± 6.74	−14.20 ± 0.23	29.10 ± 2.07	−43.07 ± 4.53
ATP Binding site						
PGG	−84.72 ± 3.26	−65.65 ± 2.84	75.80 ± 2.31	−13.31 ± 0.42	16.44 ± 1.35	−71.45 ± 4.51
ATP	−41.73 ± 0.51	−519.36 ± 6.30	495.84 ± 3.23	−6.55 ± 0.22	50.60 ± 6.81	−21.21 ± 6.72

^a^ Free energy was reported from the average of three replica copies ± standard error of mean (SEM). PGG: pentagalloyl glucose; ATP: adenosine triphosphate; $\Delta E_{\mathrm{vdw}}$: van der Waals energy; $\Delta E_{\mathrm{elect}}$: electrostatic energy; $\Delta G_{\mathrm{polar}}$: free energy of polar solvation; $\Delta G_{\mathrm{nonpolar}}$: free energy of non-polar solvation; −T∆S: conformational entropy after ligand binding; $\Delta G_{\mathrm{bind}}$: total binding free energy; binding free energy was reported as mean ± standard error of mean (SEM).

## Data Availability

Data is contained within the article.

## Supplementary Materials

The following supporting information can be downloaded at: https://www.mdpi.com/article/10.3390/ph16091192/s1, Figure S1: P-glycoprotein (P-gp) structure consists of two transmembrane domains, each including six helices that transverse the cellular membrane. In addition, it contains two ATP-binding regions (nucleotide-binding domains, NBDs) within the cell; Figure S2: Tariquidar superimposed between docked and co-crystalized ligands at the substrate binding site for docking protocol validation (PDB ID: 7A6E). The result shows an RMSD value of 1.465 Å; Figure S3: Superimposed between the docked and co-crystalized ATP at the ATP binding site for docking protocol validation (PDB ID: 6C0V). The result shows an RMSD value of 0.766 Å; Figure S4: Three-dimensional structures of PGG, tariquidar, verapamil, and ATP together with the best fit of these structures; Figure S5: Three-dimensional structures of protein–ligand complexes and free energy landscape (FEL) of three replica copies of verapamil at the substrate binding site of P-gp; Figure S6: Three-dimensional structures of protein–ligand complexes and free energy landscape (FEL) of three replica copies of tariquidar at the substrate binding site of P-gp; Figure S7: Three-dimensional structures of protein–ligand complexes and free energy landscape (FEL) of three replica copies of PGG at the substrate binding site of P-gp; Figure S8: Three-dimensional structures of protein–ligand complexes and free energy landscape (FEL) of three replica copies of ATP at the ATP binding site of P-gp; Figure S9: Three-dimensional structures of protein–ligand complexes and free energy landscape (FEL) of three replica copies of PGG at the ATP binding site of P-gp; Figure S10: The uncropped Western blot images corresponding to Figure 3a.
