# Supplementary material for: Pentagalloyl Glucose-Targeted Inhibition of P-Glycoprotein and Re-Sensitization of Multidrug-Resistant Leukemic Cells (K562/ADR) to Doxorubicin: In Silico and Functional Studies
Source: Pharmaceuticals (Basel). 2023 Aug 22;16(9):1192. doi: 10.3390/ph16091192 (PMC10535865; doi:10.3390/ph16091192)
Supplement: Supplementary file 1 [file pharmaceuticals-16-01192-s001.zip › pharmaceuticals-2527984-supplementary.pdf]

Supplementary Material

Pentagalloyl glucose targeted inhibition of P-glycoprotein and re-sensitization of multidrug-resistant leukemic cells (K562/ADR) to doxorubicin: in silico and functional studies

Nathupakorn Dechsupa[1,2], Nopawit Khamto[3,4], Pornthip Chawapun[3,4,5], Sadanon Siriphong[3,4,5], Phattarawadee Innuan[1,2], Authaphinya Suwan[1,2], Thitiworada Luangsuep[2], Nichakorn Photilimthana[2], Witchayaporn Maita[2], Rossarin Thanacharttanatchaya[2], Padchanee Sangthong[3], Puttinan Meepowpan[3,6,7], Chatchanok Udomtanakunchai[2], Jiraporn Kantapan[1,2*]

[1]Molecular Imaging and Therapy Research Unit, Faculty of Associated Medical Sciences, Department Radiologic Technology, Chiang Mai University, Chiang Mai 50200, Thailand

[2] Faculty of Associated Medical Sciences, Department Radiologic Technology, Chiang Mai University, Chiang Mai 50200, Thailand

[3]Faculty of Science, Department of Chemistry, Chiang Mai University, Chiang Mai 50200, Thailand

[4]Graduate School, Chiang Mai University, Chiang Mai 50200, Thailand

[5]Interdisciplinary Program in Biotechnology, Graduate School, Chiang Mai University, Chiang Mai 50200, Thailand

[6]Center of Excellence in Materials Science and Technology, Chiang Mai University, Chiang Mai 50200, Thailand

[7]Center of Excellence for Innovation in Chemistry (PERCH-CIC), Faculty of Science, Chiang Mai University, Chiang Mai 50200, Thailand

***Correspondence:** Jiraporn Kantapan: jiraporn.kan@cmu.ac.th

Supplementary Materials: Figure S1: P-glycoprotein (P-gp) structure consists of two transmembrane domains, each including six helices that transverse the cellular membrane. In addition, it contains two ATP-binding regions (nucleotide-binding domains, NBDs) within the cell; Figure S2: Tariquidar superimposed between docked and co-crystalized ligands at the substrate binding site for docking protocol validation (PDB ID: 7A6E). The result showed the RMSD value of 1.465 Å; Figure S3: Superimposed between the docked and co-crystalized ATP at the ATP binding site for docking protocol validation (PDB ID: 6C0V). The result showed the RMSD value of 0.766 Å; Figure S4: 3-Dimenstional structures of PGG, tariquidar, verapamil and ATP togerther with the best fit of these structures; Figure S5: 3D struc-tures of protein-ligand complex and free energy landscape (FEL) of three replica copies of verapamil at substrate binding site of P-gp; Figure S6: 3D structures of protein-ligand complex and free energy landscape (FEL) of three replica copies of tariquidar at substrate binding site of P-gp; Figure S7: 3D structures of protein-ligand complex and free energy landscape (FEL) of three replica copies of PGG at substrate binding site of P-gp; Figure S8: 3D structures of protein-ligand complex and free energy landscape (FEL) of three replica copies of ATP at ATP binding site of P-gp; Figure S9: 3D structures of protein-ligand complex and free energy landscape (FEL) of three replica copies of PGG at ATP binding site of P-gp Figure S10: The uncropped western blot images corresponding to Figure 3a.

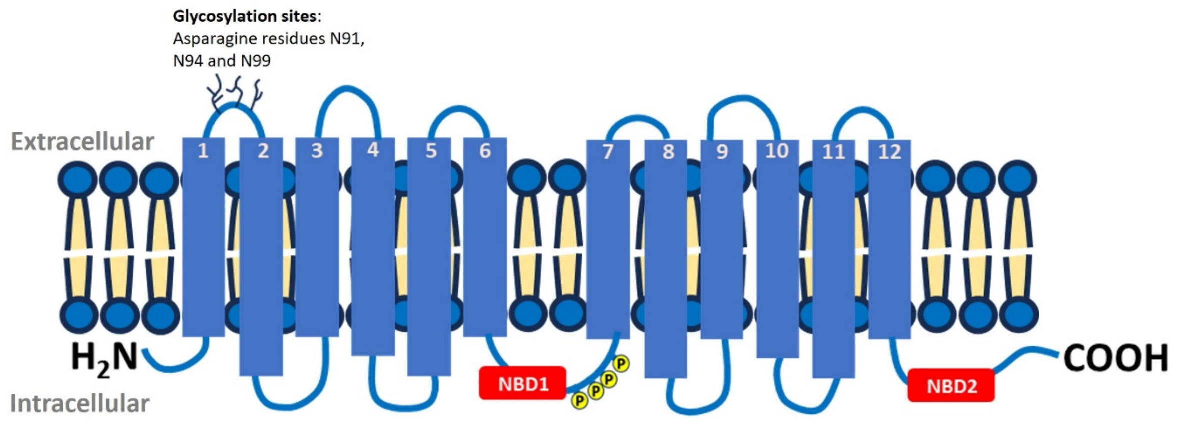

Supplementary Figure S1. P-glycoprotein (P-gp) structure consists of two transmembrane domains, each including six helices that transverse the cellular membrane. In addition, it contains two ATP-binding regions (nucleotide-binding domains, NBDs) within the cell

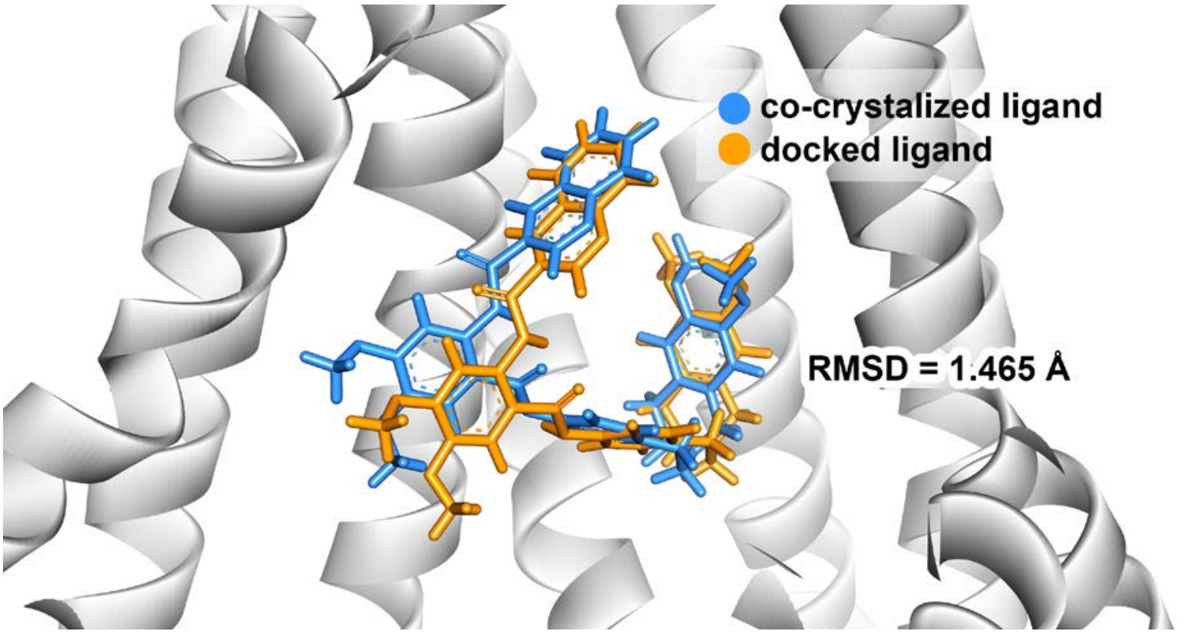

Supplementary Figure S2. Tariquidar superimposed between docked and co-crystalized ligands at the substrate binding site for docking protocol validation (PDB ID: 7A6E). The result showed the RMSD value of 1.465 Å.

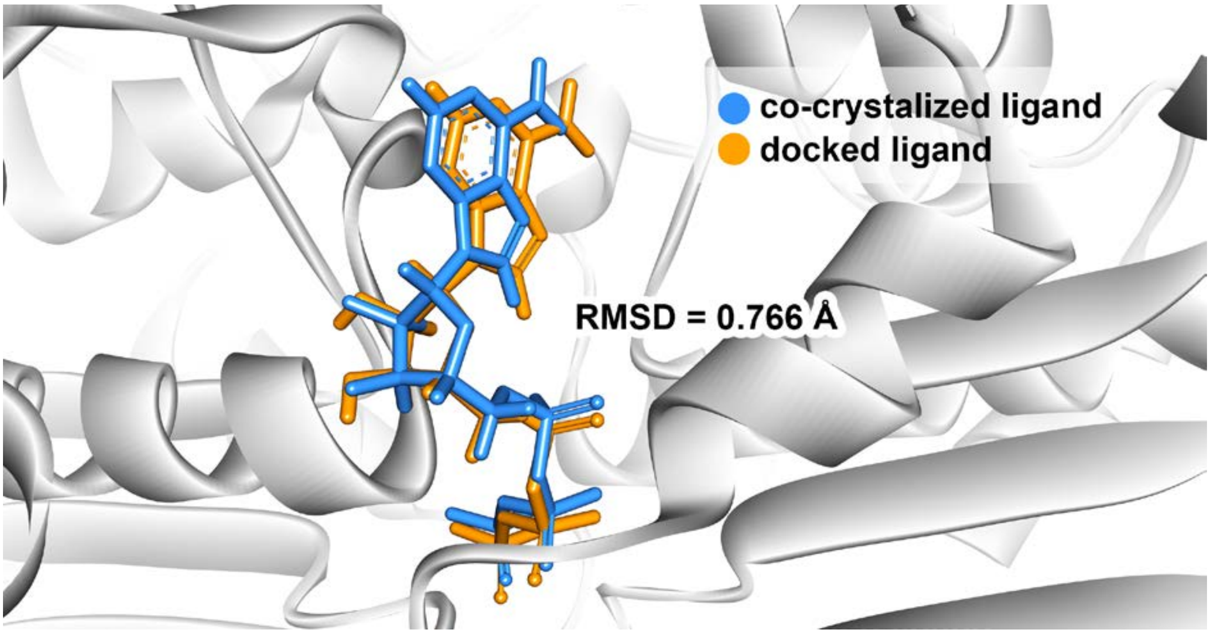

Supplementary Figure S3. Superimposed between the docked and co-crystalized ATP at the ATP binding site for docking protocol validation (PDB ID: 6C0V). The result showed the RMSD value of 0.766 Å.

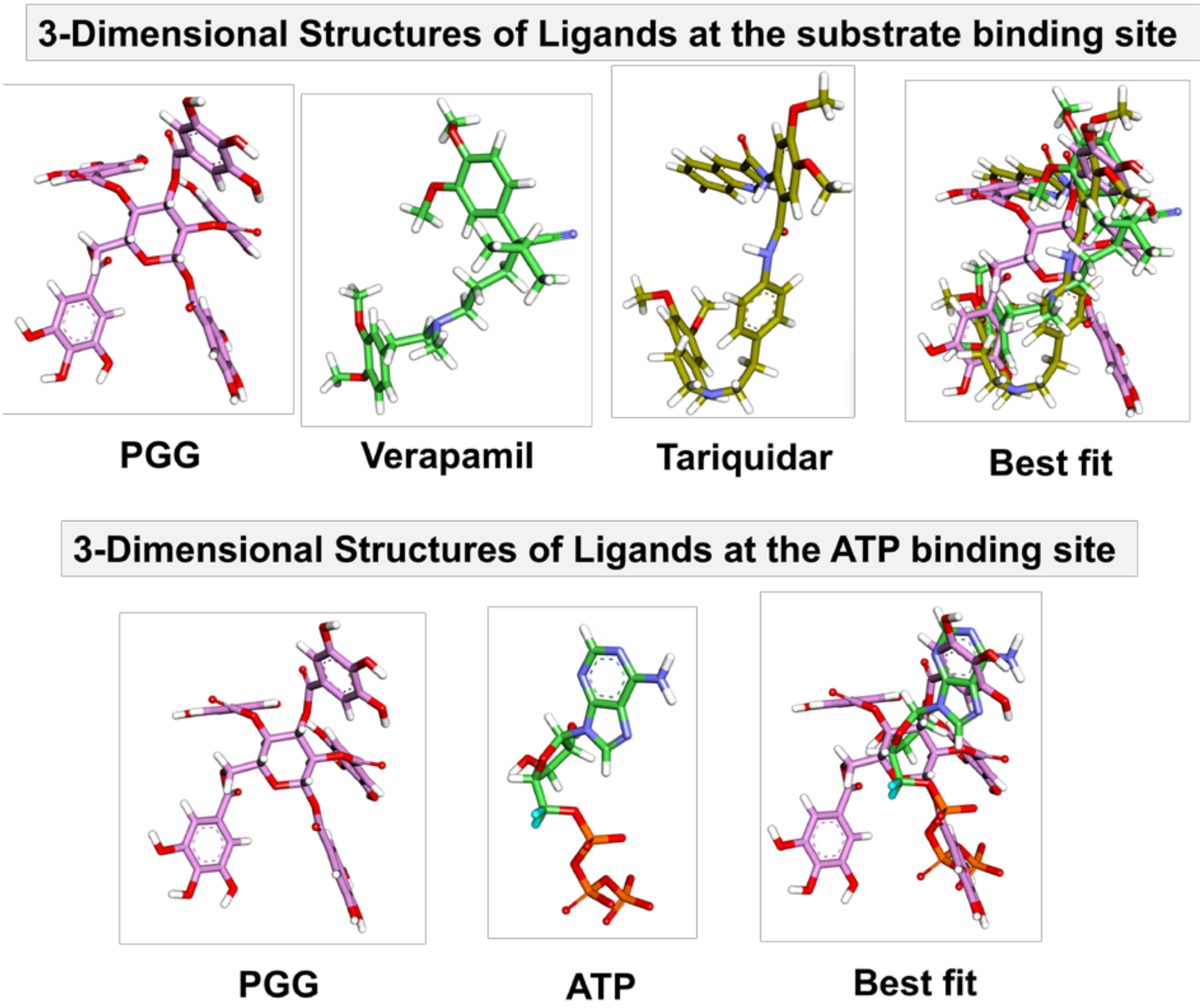

Supplementary Figure S4. 3-Dimenstional structures of PGG, tariquidar, verapamil and ATP togerther with the best fit of these structures.

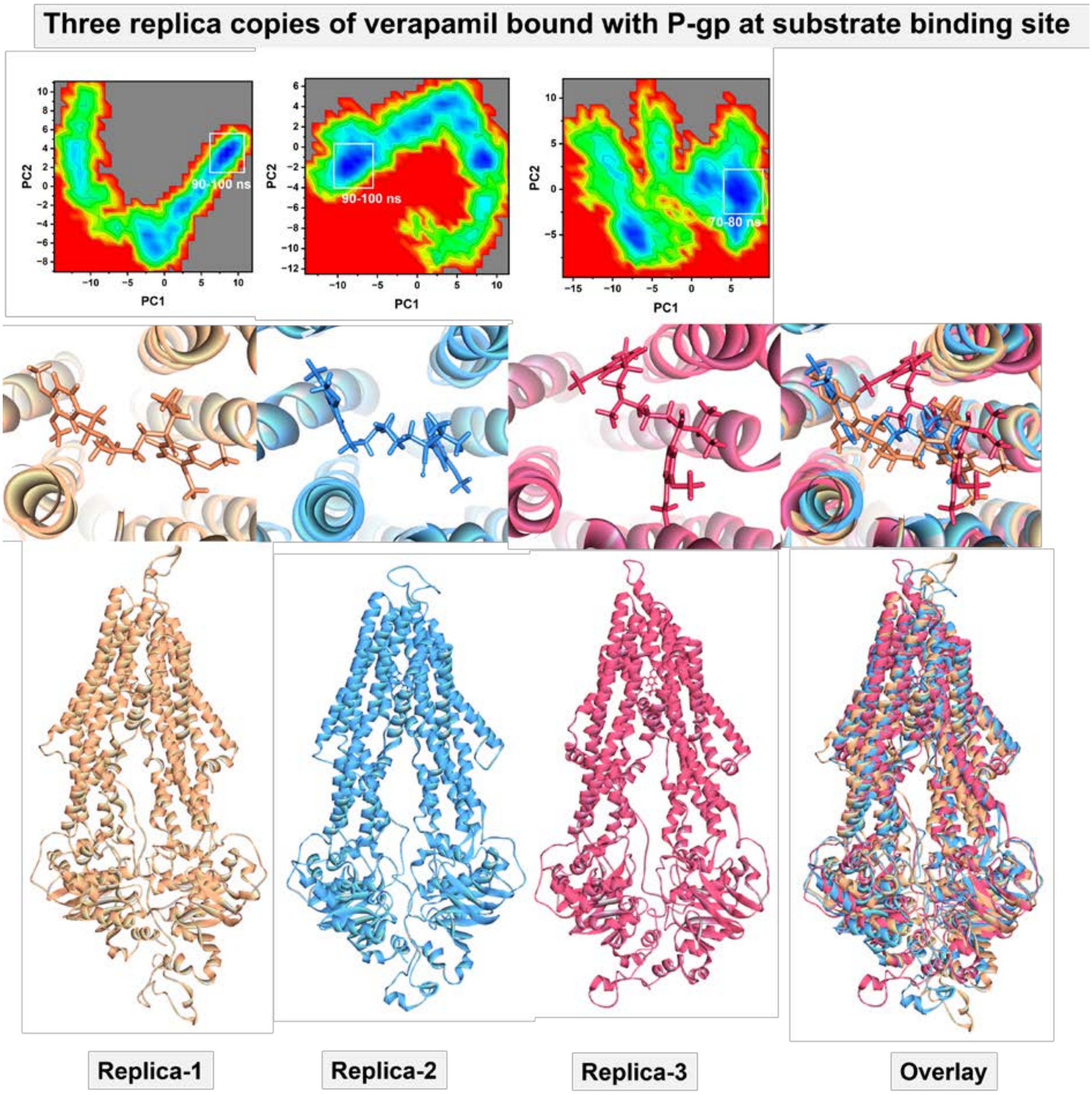

Supplementary Figure S5. 3D structures of protein-ligand complex and free energy landscape (FEL) of three replica copies of verapamil at substrate binding site of P-gp

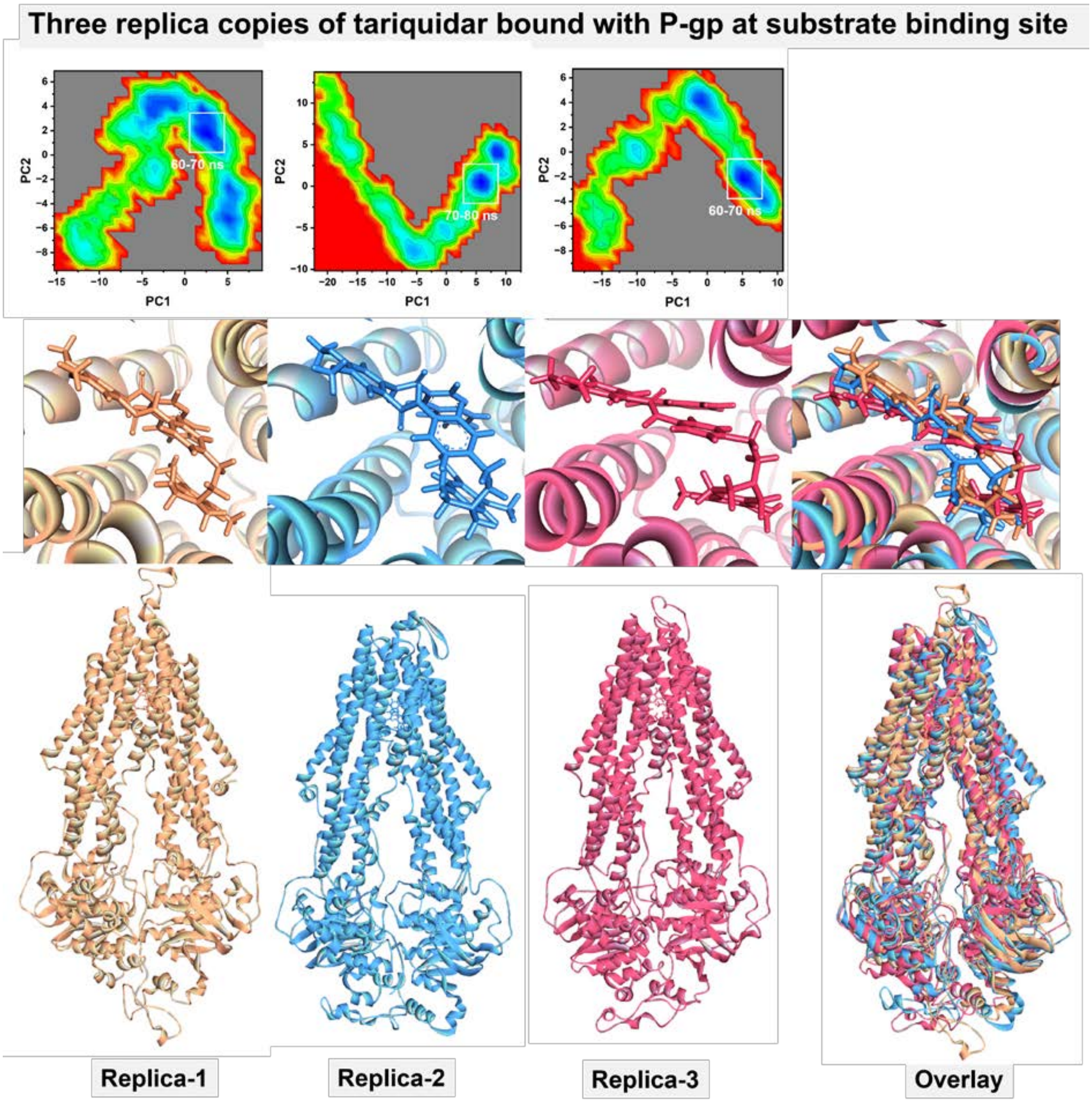

Supplementary Figure S6. 3D structures of protein-ligand complex and free energy landscape (FEL) of three replica copies of tariquidar at substrate binding site of P-gp

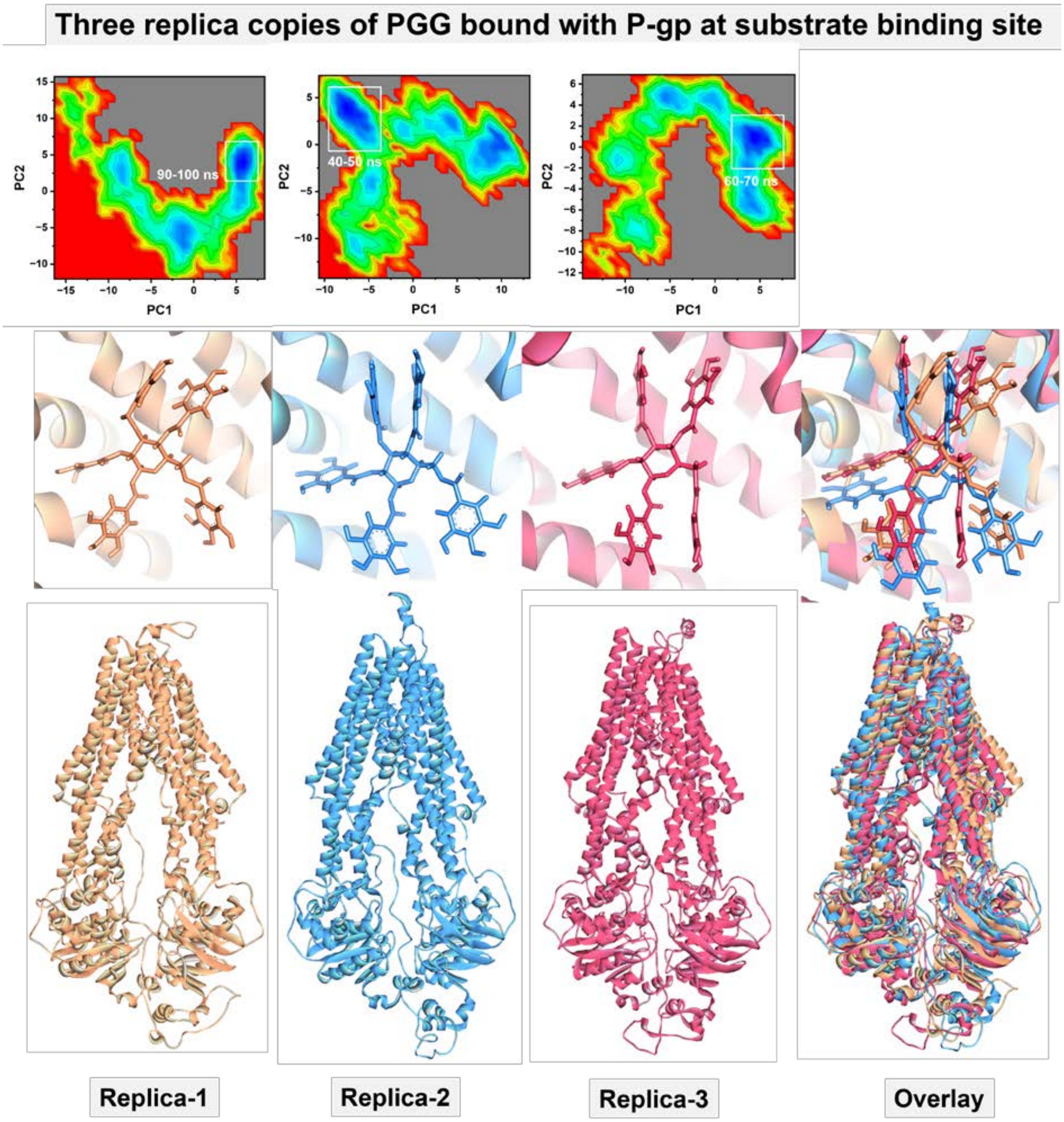

Supplementary Figure S7. 3D structures of protein-ligand complex and free energy landscape (FEL) of three replica copies of PGG at substrate binding site of P-gp

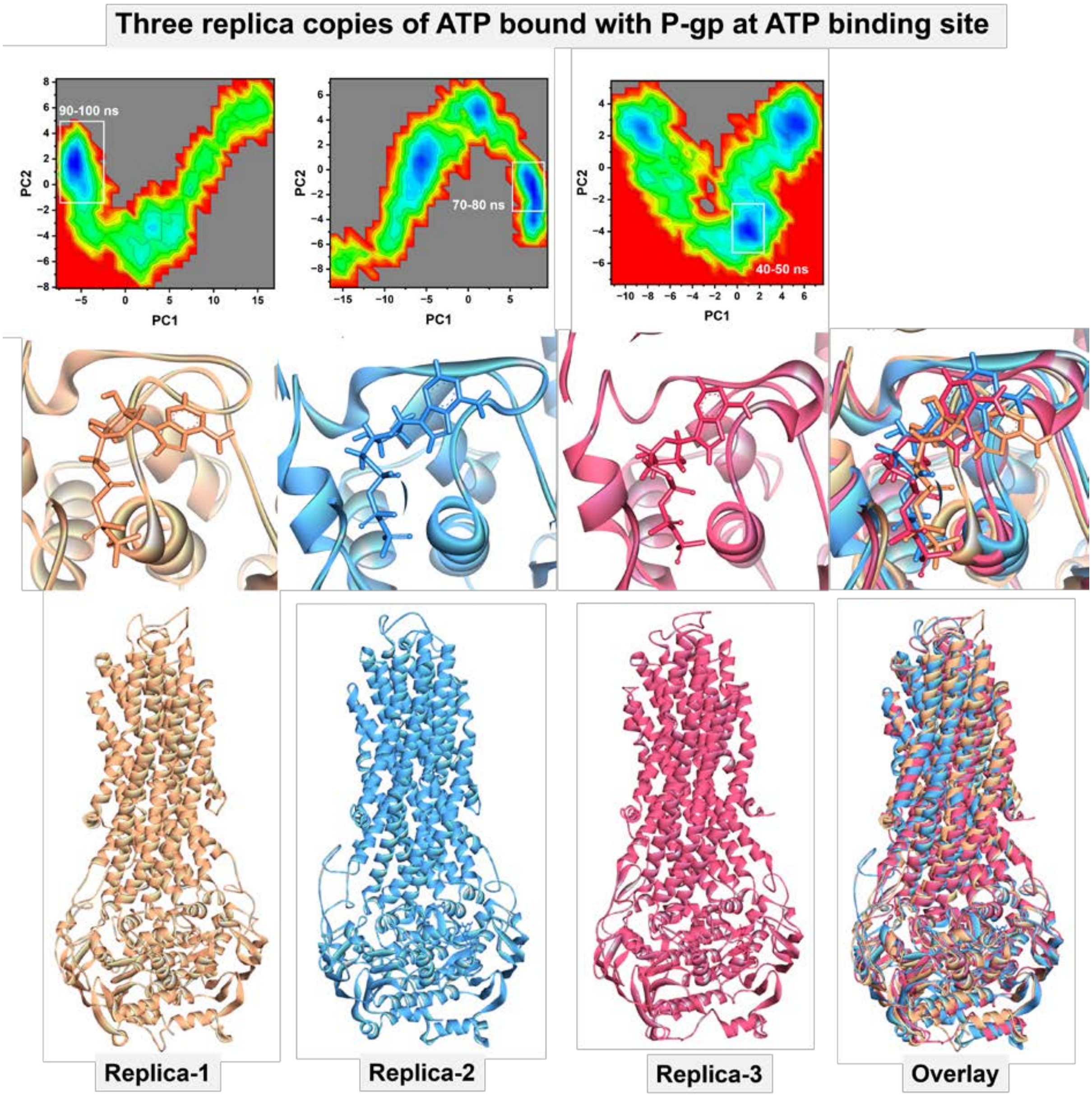

Supplementary Figure S8. 3D structures of protein-ligand complex and free energy landscape (FEL) of three replica copies of ATP at ATP binding site of P-gp

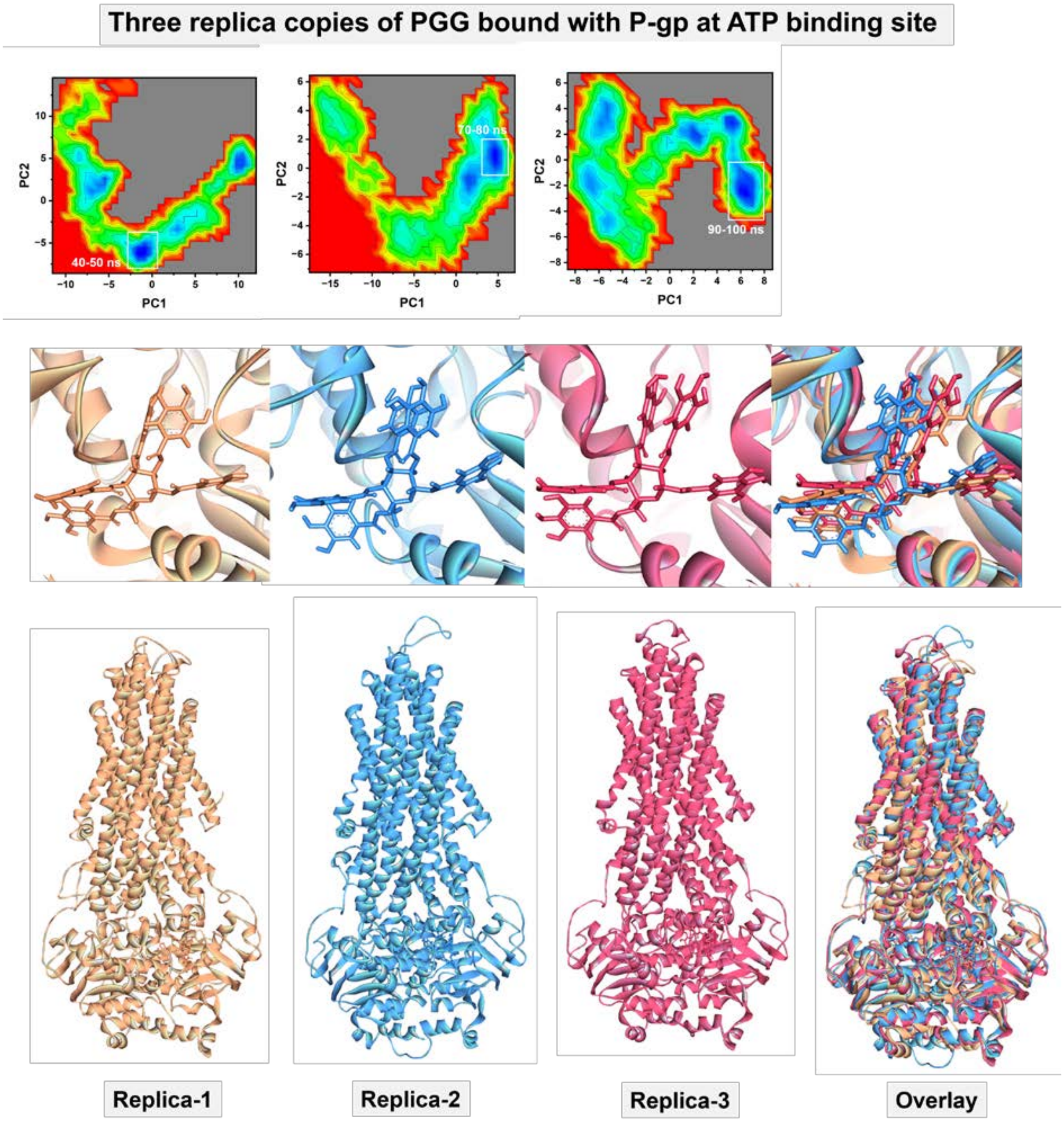

Supplementary Figure S9. 3D structures of protein-ligand complex and free energy landscape (FEL) of three replica copies of PGG at ATP binding site of P-gp

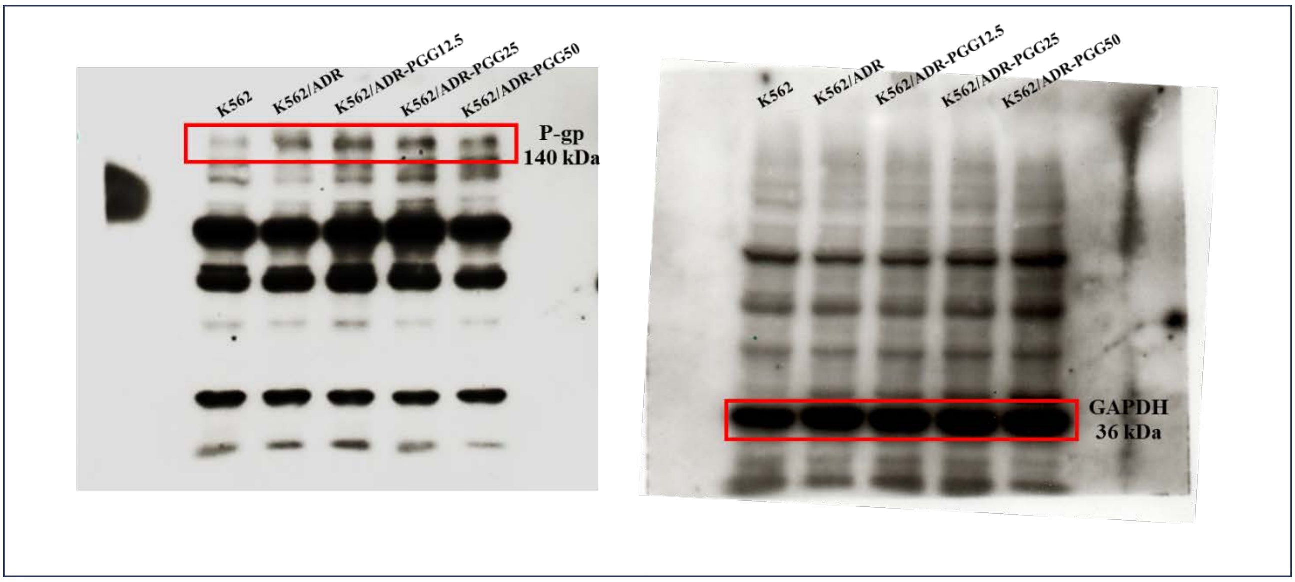

Supplementary Figure S10. The uncropped Western blot images corresponding to Figure 3a showing all the bands. Red boxes indicate the samples of interest.
